# Class Id ribonucleotide reductase utilizes a Mn_2_(IV,III) cofactor and undergoes large conformational changes on metal loading

**DOI:** 10.1007/s00775-019-01697-8

**Published:** 2019-08-14

**Authors:** Inna Rozman Grinberg, Sigrid Berglund, Mahmudul Hasan, Daniel Lundin, Felix M. Ho, Ann Magnuson, Derek T. Logan, Britt-Marie Sjöberg, Gustav Berggren

**Affiliations:** 1 Department of Biochemistry and Biophysics, Stockholm University, Stockholm, Sweden; 2 Department of Chemistry, Ångström Laboratory, Uppsala University, Uppsala, Sweden; 3 Department of Biochemistry and Structural Biology, Lund University, Lund, Sweden

**Keywords:** Ribonucleotide reductase, Dimanganese cofactor, Radicals, Electron paramagnetic resonance, X-ray crystallography, Phylogeny

## Abstract

Outside of the photosynthetic machinery, high-valent manganese cofactors are rare in biology. It was proposed that a recently discovered subclass of ribonucleotide reductase (RNR), class Id, is dependent on a Mn_2_(IV,III) cofactor for catalysis. Class I RNRs consist of a substrate-binding component (NrdA) and a metal-containing radical-generating component (NrdB). Herein we utilize a combination of EPR spectroscopy and enzyme assays to underscore the enzymatic relevance of the Mn_2_(IV,III) cofactor in class Id NrdB from *Facklamia ignava*. Once formed, the Mn_2_(IV,III) cofactor confers enzyme activity that correlates well with cofactor quantity. Moreover, we present the X-ray structure of the apo- and aerobically Mn-loaded forms of the homologous class Id NrdB from *Leeuwenhoekiella blandensis*, revealing a dimanganese centre typical of the subclass, with a tyrosine residue maintained at distance from the metal centre and a lysine residue projected towards the metals. Structural comparison of the apo- and metal-loaded forms of the protein reveals a refolding of the loop containing the conserved lysine and an unusual shift in the orientation of helices within a monomer, leading to the opening of a channel towards the metal site. Such major conformational changes have not been observed in NrdB proteins before. Finally, in vitro reconstitution experiments reveal that the high-valent manganese cofactor is not formed spontaneously from oxygen, but can be generated from at least two different reduced oxygen species, i.e. H_2_O_2_ and superoxide (O_2_^·−^). Considering the observed differences in the efficiency of these two activating reagents, we propose that the physiologically relevant mechanism involves superoxide.

**Electronic supplementary material:**

The online version of this article (10.1007/s00775-019-01697-8) contains supplementary material, which is available to authorized users.

## Introduction

Ribonucleotide reductase (RNR) is the only enzyme capable of de novo synthesis of deoxyribonucleotides (dNTPs). As such, it is an essential enzyme found in all species apart from a few intracellular parasites, and it is even encoded in certain dsDNA viruses to ensure ready access to DNA building blocks. RNRs form an enzyme family currently comprising three different classes and several subclasses. All RNRs share a common reaction mechanism, in which a cysteine radical induces the reduction of ribonucleotides, but they differ in the way the radical mechanism is initiated [1–5]. Class I RNRs consist of a substrate-binding component (NrdA or α) and a radical-generating component (NrdB or β). Generally, the β-subunit is dimeric (β_2_) and the active complex is a heterotetramer (α_2_β_2_) in bacteria and a heterooctamer (α_6_β_2_) in eukaryotes.

The evolutionary pressure experienced by many organisms under metal-limiting conditions is clearly reflected in RNR. This has resulted in a range of class I subclasses, featuring a rich variety of radical cofactors in the NrdB component stabilizing an electron hole required to initiate catalysis. In eukaryotic RNRs and several bacterial class I RNRs, collectively denoted class Ia, the NrdB component contains a stable tyrosyl radical in the vicinity of a diferric metal centre (Fe_2_(III,III)-Y^·^). Similarly, one bacterial subclass denoted class Ib contains a tyrosyl radical in the vicinity of a dimanganic centre (Mn_2_(III,III)-Y^·^). In one group denoted class Ic, a mutation in the radical-carrying tyrosine is accompanied by a mixed valent Mn(IV)Fe(III) metal centre that harbours the unpaired electron [6, 7]. More recently, two novel subclasses, denoted class Id and Ie, have been reported. In the case of class Ie, the metal cofactor is replaced with a single 3,4-dihydroxyphenylalanine (DOPA) organic radical [8, 9]. Conversely, class Id RNRs from *Facklamia ignava*, *Leeuwenhoekiella blandensis* and *Flavobacterium johnsoniae* are all proposed to function without an organic radical, and instead utilize a mixed valent Mn_2_(IV,III) metal centre [10–12]. However, this remains to be firmly established. The mentioned class Id RNRs belongs to the phylogenetic RNR subclass NrdAi/NrdBi [11]. Still, their NrdB domain structures differ. The *F. ignava* NrdB has a unique domain structure comprising an N-terminal glutaredoxin (Grx) domain, followed by an ATP-cone domain and a radical-generating domain [12], whereas the *L. blandensis* NrdB consists of an N-terminal ATP-cone domain followed by the radical-generating domain [11] and the *F. johnsoniae* NrdB has no additional domains [10].

High-valent Mn cofactors are rare in biology and have only been observed in a limited number of enzymes [13, 14]. More specifically, a Mn_2_(IV,III) dimer has been detected as a transient intermediate during the assembly of the aforementioned Mn_2_(III,III)-Y^·^ RNR cofactor and in “superoxidized” Mn-catalase [15, 16]. To date such high-valent manganese species have only proven to be enzymatically relevant in the case of the oxygen evolving complex, the tetranuclear manganese cluster constituting the active site of photosystem II [17, 18]. Herein we show that class Id RNR from *F. ignava* allows for the preparation of a homogenous Mn_2_(IV,III) manganese population lacking traces of other EPR signals, which has previously not been observed for other class Id RNRs. This has allowed us to verify that the activity is not only highly dependent on the presence of manganese, we also utilize a combination of EPR spectroscopy and enzyme assays to demonstrate the enzymatic relevance specifically of the Mn_2_(IV,III) cofactor. Moreover, a comparison of the X-ray structure of the apo- and manganese-loaded forms of the homologous class Id NrdB protein from *L. blandensis* reveals a striking refolding of a loop in the vicinity of the metal site. Finally, in vitro reconstitution experiments show that the high-valent manganese dimer can be generated from at least two different oxidants, i.e. H_2_O_2_ and superoxide (O_2_^·−^). Considering the observed differences in efficiency of these two activating reagents, we propose that the relevant mechanism in vivo involves superoxide.

## Results

### Class Id enzyme activity correlates with Mn content

As we have previously reported, heterologous expression of the class Id NrdB protein from *F. ignava* (subclass NrdBi [13]) in the presence of excess Mn^2+^ ions generates a form of the protein featuring a multiline EPR signal attributable to a Mn_2_(IV,III) cofactor [12]. To further probe the metal cofactor in this protein and elucidate its assembly mechanism we isolated a series of different forms of the *F. ignava* NrdBi protein. The full-length, wild-type (WT-NrdB) form of the protein was prepared as reference material, and for solubility and stability purposes, we prepared a deletion mutant, NrdB∆169, in which the N-terminal Grx and ATP-cone domains were removed. When WT-NrdB was expressed in standard Luria–Bertani (LB) media, the isolated protein contained low levels of metals, primarily Fe (Table 1), as quantified by total-reflection X-ray fluorescence (TXRF). Conversely, expression of the full-length protein in Mn^2+^-enriched media resulted in NrdB samples containing 1.49 Mn/NrdB (WT-NrdB^Mn^). Similarly, we expressed the truncated protein in Mn^2+^-enriched media (NrdB∆169^Mn^) or in the presence of the metal chelator EDTA (NrdB∆169^apo^). Isolated NrdB∆169^Mn^ contained 1.8 Mn ions/NrdB further supporting the notion of dinuclear metal site, whereas NrdB∆169^apo^ was isolated with a negligible amount of Mn but some Fe (Table 1).

**Table 1:** Metal content and specific activity observed for different metals containing *F. ignava* NrdB forms

*F. ignava* NrdBi form	Expression medium	Mn content (mol/mol protein)	Fe content (mol/mol protein)	Specific activity (nmol mg^−1^ min^−1^)
WT-NrdB	LB	0.14 ± 0.02	0.79 ± 0.05	409 ± 145
WT-NrdB^Mn^	LB + 0.5 mM Mn(CH_3_CO_2_)_2_	1.49 ± 0.16	0.04 ± 0.04	4083 ± 210
NrdB∆169^apo^	LB + 0.5 mM EDTA	0.01 ± 0.002	0.22 ± 0.02	341 ± 44
NrdB∆169^Mn^	LB + 0.5 mM Mn(CH_3_CO_2_)_2_	1.8 ± 0.003	0.01 ± 0.003	3626 ± 621

Standard deviations are based on three measurements

**Fig. 1:**
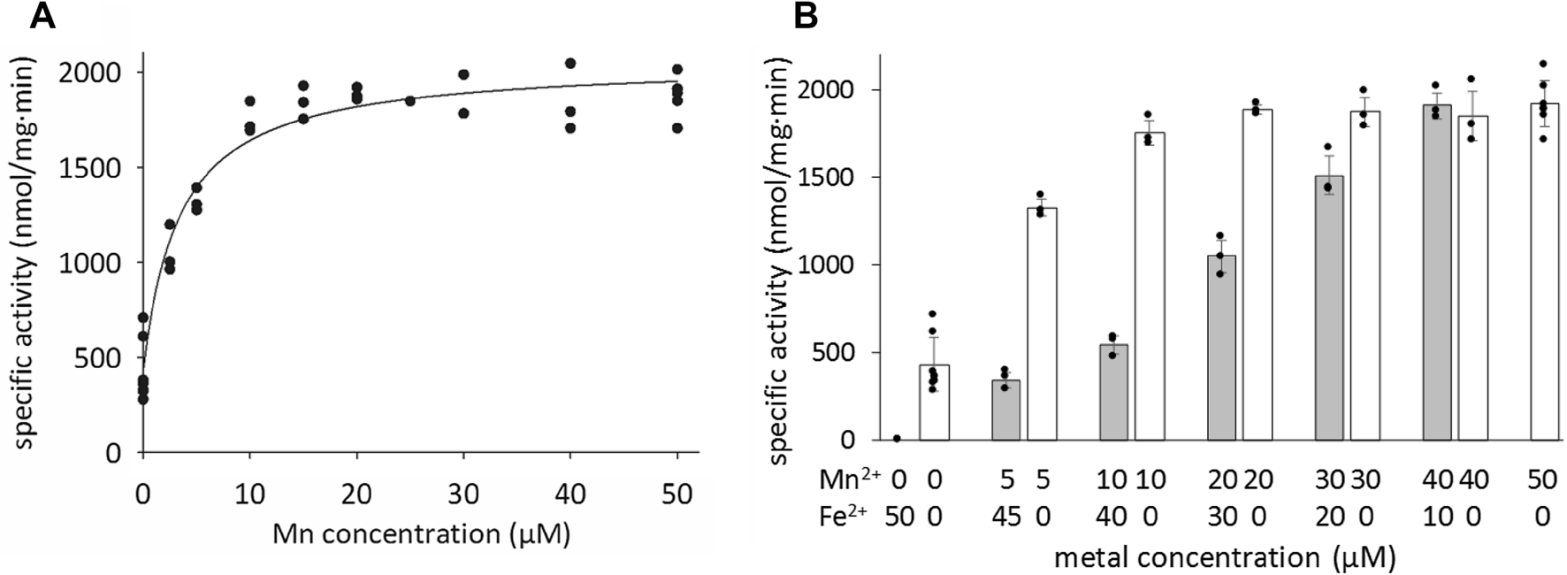
Specific activity (nmol dCDP∙mg_NrdB_^−1^ min^−1^) observed for *F. ignava* NrdB∆169^apo^ following reconstitution with increasing amounts of Mn^2+^ (A; B*, white bars*) and in combination with Fe^2+^ (B, *grey bars*). Prior to activity assays, 10 µM NrdB∆169^apo^ was incubated with the indicated amounts of Mn(CH_3_CO_2_)_2_ or in combination with Fe(SO_4_)·7H_2_O for 1 h at room temperature on the bench without any addition of oxidant. The metal reconstituted protein was then added to the reaction mixture (containing 2.5 µM NrdA) to reach a final concentration of 0.25 µM, and assays were performed as described in “Materials and methods”. The apparent *K*_L_ value was determined through the plot of *v* = (*V*_max_·[Mn])/(*K*_L_ + [Mn]) (solid black line)

The capacity of these four different forms of *F. ignava* NrdB to support catalysis was tested in CDP reduction assays in the presence of excess NrdA and with the chemical DTT as sacrificial reductant (Table 1). Both WT-NrdB^Mn^ and NrdB∆169^Mn^ provided high specific activity (WT-NrdB^Mn^ 4083 ± 210 nmol dCDP formed/min mg and NrdB∆169^Mn^ 3626 ± 621 nmol dCDP formed/min mg, corresponding to *k*_cat_ values of 8.2 s^−1^ and 4.9 s^−1^, respectively, for the enzymatically relevant β_2_ form). WT-NrdB with 10% Mn content compared to WT-NrdB^Mn^ had a correspondingly lower specific activity of 409 ± 145 nmol CDP formed/min mg. The fact that also NrdB∆169^apo^ had some residual activity (341 ± 44 nmol dCDP formed/min mg), which was completely inhibited by incubation with excess Fe^2+^ (Fig. 1b), suggests that, at the the low concentration used in the assay, the protein may become activated by traces of manganese in the assay mixture. In combination, these observations clearly support the importance of the manganese cofactor for catalysis, in good agreement with earlier reports on NrdBi from not only *F. ignava*, but also *L. blandensis* and *F. johnsoniae* [10–12].

### Activity assays in the presence of Mn^2+^ generate active NrdB

To further probe the metal dependence of the *F. ignava* RNR, a series of assays was performed in which NrdA was mixed with NrdB∆169^apo^ in combination with varying amounts of Mn^2+^ and/or Fe^2+^ salts. As seen in Fig. 1a, the addition of Mn^2+^ to the assay had a strong activating effect. Close to maximum enzyme activity was obtained after the addition of 1 equivalent of Mn^2+^/NrdB monomer. The apparent *K*_L_ for Mn^2+^ was 3.4 µM and the calculated *V*_max_ was 2056 nmol dCDP formed/min mg. The presence of Fe^2+^ in the Mn^2+^-containing samples inhibited the activity (Fig. 1b). However, in the presence of excess Mn^2+^ the inhibitory effect of Fe^2+^ became negligible.

### Signatures of the NrdBi subclass

**Fig. 2:**
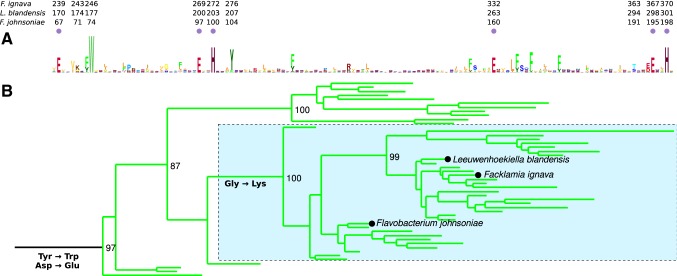
Sequence logo and phylogeny of NrdBi. **a** Sequence logo generated with Skylign [19] from aligned NrdBi sequences. Amino acid coordinates for *F. ignava*, *L. blandensis* and *F. johnsoniae* NrdBi sequences are indicated. Purple circles indicate metal ligating amino acids and arrows denote NrdBi-specific mutations indicated in **b**. **b** Excerpt from NrdBi maximum likelihood phylogeny (for the full tree with outgroup in nexml format, see 10.17045/sthlmuni.8386652.v1). The boxed area indicates the subgroup in which a Lys nearby the conserved Trp and the N-terminal metal ligating residues is conserved. “Tyr → Trp”, “Asp → Glu” and “Gly → Lys” indicate where key substitutions presumably occurred. The full phylogeny is shown in figure S1

NrdBi proteins from three different bacteria have been studied: *L. blandensis* NrdBi that has an N-terminal ATP-cone domain [11], *F. ignava* NrdBi that has an N-terminal glutaredoxin (Grx) domain followed by an ATP-cone domain [12], and *F. johnsoniae* NrdBi that lacks additional fusions [10]. The NrdBi subclass is most closely related to the NrdBk subclass, for which a representative enzyme remains to be isolated and characterized. Distinct differences between NrdBk and NrdBi are two striking mutations, a change of the most N-terminal metal ligating residue from Asp to Glu239 (in *F. ignava* numbering) and a change of a strictly conserved Tyr (of unknown function) in NrdBk to a strictly conserved Trp246 (in *F. ignava* numbering) in NrdBi (Fig. 2). Furthermore, a mutation of a Gly to a conserved Lys243 (in *F. ignava* numbering) occurred at a later stage, and is hence conserved only in a subgroup of NrdBi (Fig. 2). All three hitherto studied NrdBi proteins belong to this subgroup, with a conserved lysine three residues upstream of the conserved tryptophan (Fig. 2). Considering these similarities and the shared dependence on manganese for activity in the NrdBi proteins from *F. ignava, L. blandensis* and *F. johnsoniae*, it is highly likely that all three share the same type of cofactor.

### Crystal structures of the apo- and Mn^2+^-loaded forms of NrdBi from *L. blandensis*

For structure determination and interpretation, the homologous NrdBi protein from *L. blandensis* (*Lb*NrdB) was used as a proxy for the protein from *F. ignava,* due to difficulties in crystallizing the latter (Table 2). The two proteins have 62% overall sequence identity in the core metal-binding domain, and all functionally important residues in the metal-binding site are completely conserved. In addition, we earlier showed that the *Lb*NrdB protein is enzymatically functional in heterologous mixtures with NrdA from *F. ignava* [12]. The structure of full-length *Lb*NrdB has already been presented (PDB ID 5OLK), albeit in a functionally irrelevant Ca^2+^-loaded form that originated from the crystallization conditions [11]. Here, we crystallized and solved the structures of an N-terminally truncated form of *Lb*NrdB lacking the ATP-cone domain, *Lb*NrdBΔ99, in two forms: apo and aerobically Mn^2+^ loaded. This construct is equivalent to NrdB∆169 from *F. ignava.*

**Table 2:** Data processing and refinement statistics for the X-ray crystal structures

Structure	apo	Mn^2+^	Mn^2+^ anomalous
PDB code	6SF4	6SF5	–
Station	P13	P14	P14
Wavelength [Å]	0.8500	0.9763	1.8500
Space group	P2_1_2_1_2_1_		
Unit cell (Å)	*a* = 67.6 *b* = 68.2 *c* = 148.2	*a* = 57.1 *b* = 80.6 *c* = 158.2	*a* = 57.1 *b* = 80.7 *c* = 158.3
Resolution range [Å]	49.9–1.70 (1.73–1.70)	80.6–1.90 (1.94–1.90)	80.7–2.18 (2.24–2.18)
Completeness [%]	97.5 (76.2)	99.9 (99.5)	97.3 (69.5)
Total reflections	1300148 (44860)	380486 (17348)	461107 (14301)
Unique reflections	74093 (3025)	58358 (3670)	38153 (2316)
Multiplicity	17.5 (14.8)	6.5 (4.7)	12.1 (6.2)
CC_1/2_ CC_anom_	0.999 (0.538)	0.999 (0.475)	0.999 (0.902) 0.441
*R*_merge_ [%]	0.106 (2.299)	0.062 (1.137)	0.061 (0.399)
*R*_pim_ [%]	0.035 (0.854)	0.028 (0.658)	0.026 (0.246)
Mean *I*/*σ* (I)	14.5 (1.0)	13.5 (1.2)	24.1 (3.8)
Wilson B-factor [Å^2^]	32.5	35.0	33.7
R_model_ (F) [%]	0.179 (0.253)	0.187 (0.238)	0.191 (0.219)
R_free_ (F) [%]	0.195 (0.279)	0.208 (0.263)	0.228 (0.264)
Reflections used in refinement (for *R*_free_)	74037 (3669)	58284 (2895)	38063 (2073)
Average B-factors (Å^2)^	Protein: 34.3 Solvent: 41.6	Protein: 53.3 Mn^2+^: 36.0 solvent: 55.3	–
Ramachandran preferred/favoured/outliers [%]	97.1/2.9/0.0	98.6/1.4/0.0	–
Rotamer outliers (no., %)	5.0.9%	0.0.0%	–
MolProbity clash score	1.02	2.12	
Bond length rmsd from ideal [Å]	0.010	0.010	–
Bond angle rmsd from ideal [°]	0.88	0.92	

Values in parentheses are for the highest resolution shell, unless noted otherwise

**Fig. 3:**
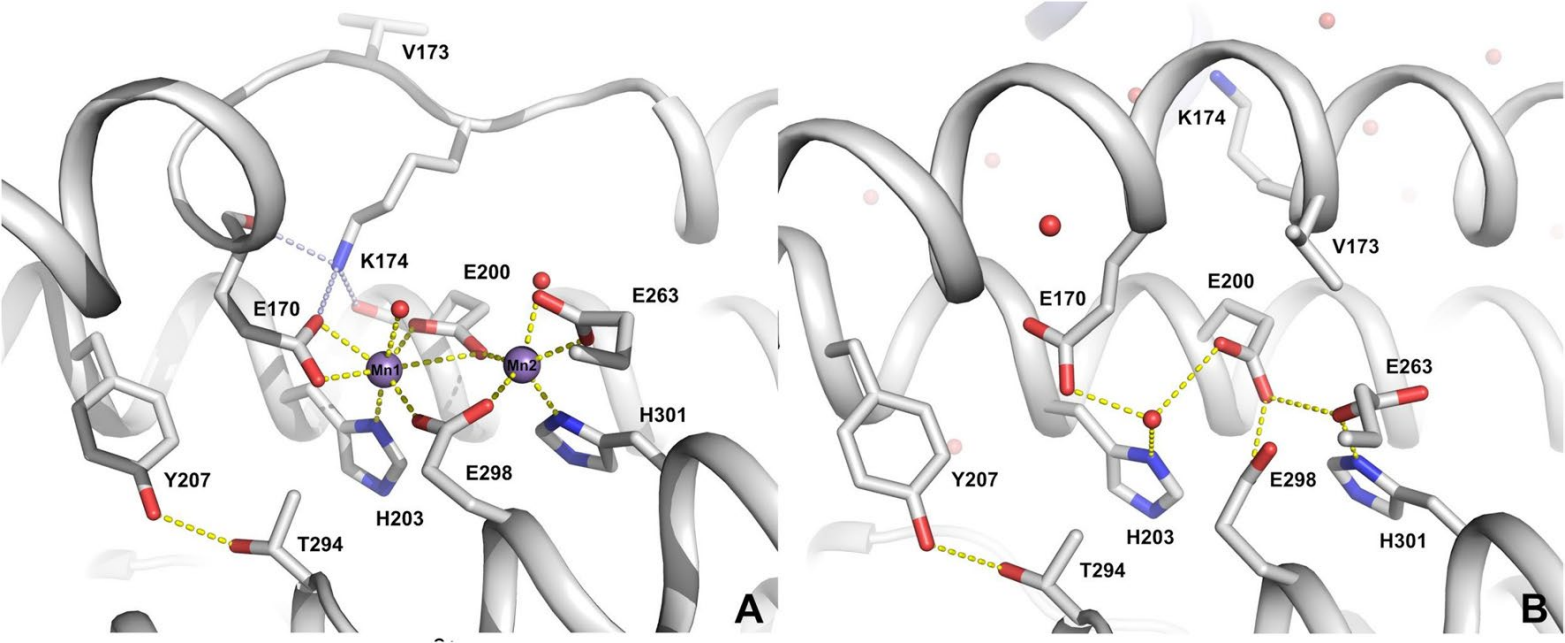
Structure of the Mn^2+^-binding site in *Lb*NrdB∆99. **a** The aerobically Mn-loaded form; **b** the apo form. Mn ions are shown as purple spheres. Coordinating ligands and other important residues are shown as sticks. Metal coordination is indicated by yellow dotted lines. Hydrogen bonds holding the side chain of Lys174 in place are shown with light-blue dotted lines. Water molecules are shown as small red spheres

Aerobically Mn^2+^-reconstituted *Lb*NrdB∆99 crystallized in space group P2_1_2_1_2_1_ with one dimer in the asymmetric unit. The overall structure is almost identical to that of the Ca^2+^-loaded form [11]. The rms deviations in Cα positions between the structures range from 0.27 to 0.52 Å for 293 atoms when all possible superpositions of chains are considered. The corresponding values for the *F. johnsoniae* class Id NrdB (PDB ID 6CWO, 45% identity) are 0.94–1.15 Å for 277–282 atoms. Anomalous difference maps at 2.2 Å resolution from data collected at a wavelength on the high-energy side of the Mn^2+^ K edge (1.85 Å) clearly confirm the identity of the metals. The four Mn^2+^ ions have different Fourier peaks ranging from 29 to 47*σ*. For comparison, the next highest peaks are at 7–9σ, from sulphur atoms in protein side chains. The Mn^2+^ ions are 3.9 Å from each other (Fig. 3a; Fig. S2). Two acidic residues, Glu200 and Glu298, bridge the ions. One manganese ion (Mn1) is heptacoordinate, having a bidentate interaction with Glu170 and monodentate interactions to Glu200, Glu298, His203 and a water molecule. The other manganese ion (Mn2) is pentacoordinate, with a bidentate interaction to Glu263 and monodentate interactions to Glu200, Glu298, His301 and a loosely bound water molecule (2.6 Å). In monomer B, Mn2 lacks coordination by the water molecule (Fig. S2). The metal coordination is very similar to that previously observed for the homologous NrdB enzyme from *F. johnsoniae* when aerobically loaded with Mn^2+^. As previously seen, a conserved lysine residue, Lys174, is projected inwards from an unusual loop that bisects helix B, and its side chain is held in place by a network of H-bonds to the carbonyl oxygen atoms of Glu170 and Glu200, as well as the Mn^2+^-coordinating side chains of the same residues (Fig. 3a). This creates an open, water-filled channel that, unusually for NrdB, positions the metal centre near the surface of the protein.

The apo form of *Lb*NrdB∆99 also crystallized in P2_1_2_1_2_1_ with one dimer in the asymmetric unit, but with different cell dimensions. In the apo form, as seen previously in NrdB subunits [20–23], the metal-coordinating residues adjust to occupy the space vacated by the metal ions and engage in a network of H-bonds to each other (Fig. 3b). Two of these H-bonds are unusually short, i.e. between Glu170 and the water molecule (2.4 Å) and between Glu263 and His301 (2.5 Å). These short distances are consistent between the two monomers. A more dramatic change between apo- and Mn^2+^-forms is seen in Lys174, which is in the apo form projected away from the metal site and into a water-filled channel between the two monomers of the dimer (Fig. 3b). This suggests that formation of the dimanganic site is accompanied by a refolding of the loop containing residues 171–174 (see below).

### Unusual conformational changes on metal loading

**Fig. 4:**
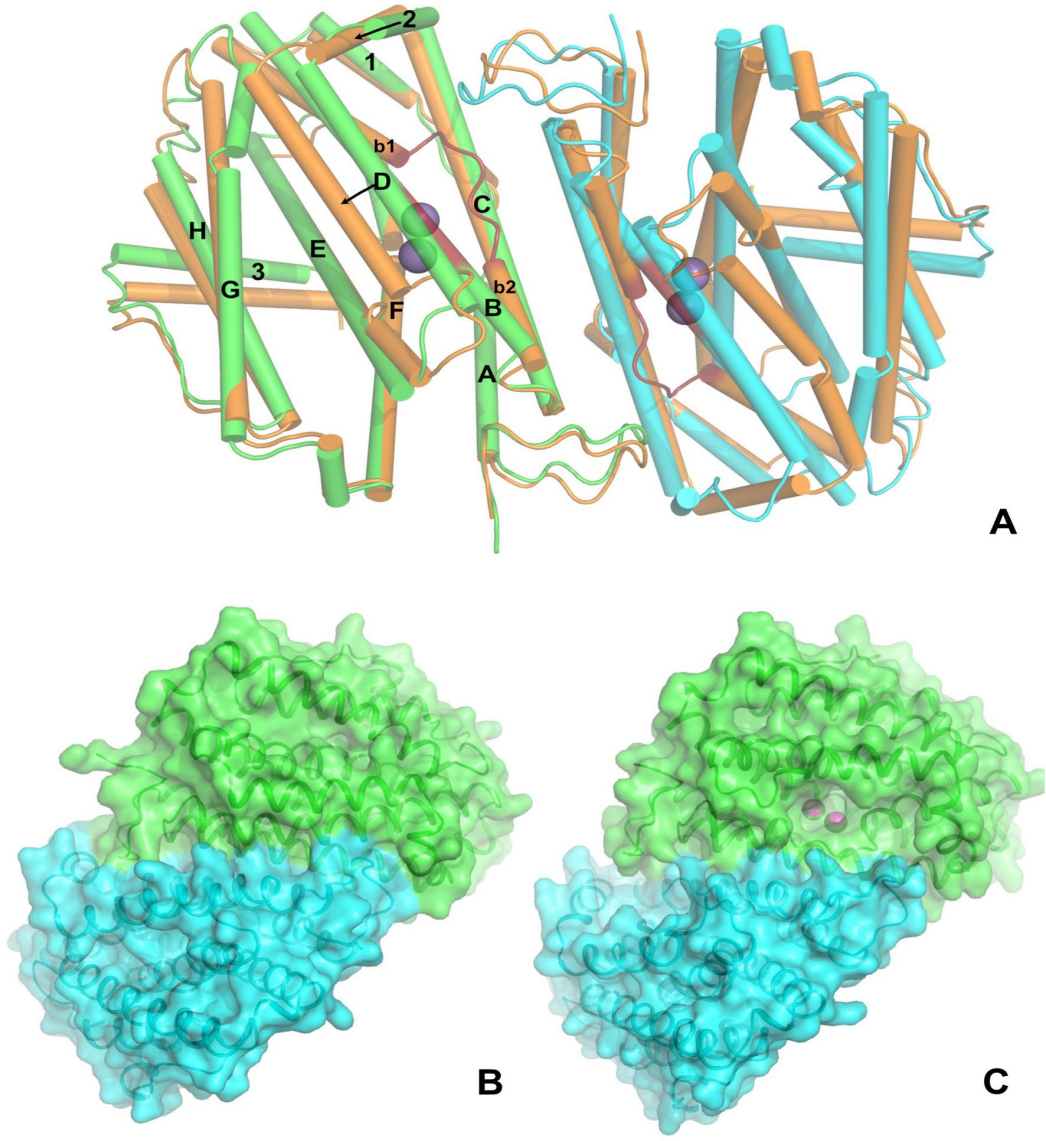
Comparison of the apo and metal-loaded forms of *Lb*NrdB∆99. **a** Shifts in the α-helices are as a result of metal loading. The Mn^2+^-containing form is coloured in green and light blue; the metal-loaded form is in one shade of orange. For clarity, α-helices are drawn as thin cylinders. Mn^2+^ ions are shown as purple spheres. The segment 170–176 in both forms containing Lys174 is coloured in a deeper shade of red. The structures are superimposed using the Cα and Cβ atoms of the Mn ligands of the left-hand monomer in both forms. **b** Surface representation of the apo form. **c** Surface representation of the metal-loaded form. Comparison of **b** and **c** clearly shows the opening up of a channel for superoxide access as a result of metal loading, which results primarily from a shift in helix **d**. The view is rotated approximately 90° clockwise with respect to panel **a**)

The metal ligands in *Lb*NrdB∆99 are found on helices B, C, E and F of the helix bundle core domain, as is usual for the ferritin superfamily [24]. The interface between monomers in the dimer consists of a four-helix bundle involving helices B and C (residues 153–183 and 187–214, respectively; Fig. 4) Helix B has 61% sequence identity between *L. blandensis* and *F. ignava* and 42% identity in a comparison of *F. johnsoniae* and the two other sequences (Fig. S3). As noted above, in the metal-loaded state, helix B is divided into two parts by an unusual central loop that projects Lys174 towards the metal site. In contrast, in the apo state, helix B is an uninterrupted α-helix, albeit that residues 177–179 near the C terminus are distorted towards 3_10_ conformation in both the apo- and Mn^2+^-states. The neighbouring Val173 is oriented towards the empty metal site, but upon metal loading it is repositioned towards the outside of the monomer, where it engages in hydrophobic interactions with Leu113 and Pro187 of the other monomer.

Conformational changes between apo- and metal-loaded states are complex and global, resulting in a surprisingly large rms deviation in Cα positions within a single monomer of 1.58 Å for all 292 atoms. We reasoned that the most objective way to compare the effect of metal loading on the protein conformation was to superimpose the crystal structures on the metal ligands, which we did using their Cα and Cβ atoms. When superimposed in this way, most of the helices in the monomer also superimpose well, including the C-terminal segment of helix B that follows the refolding segment, as well as the whole of helix C. In contrast, there is a drastic change in the register of the N-terminal part of helix B. Equivalent residues between the two forms are shifted by an average of 3.8 Å. Perhaps most remarkably, helix D (residues 227–245) moves away from a position midway between helices B and E (Fig. 4; Supplementary Movie S1), in a rigid body movement of approximately 6 Å, accompanied by an unfolding of its C-terminal end. Helix 2 follows in the same direction. In the apo form, Glu237 on helix D makes a salt bridge to Lys109 in the other monomer. Upon refolding of the middle of helix B, this salt bridge is lost (Fig. S3). Simultaneously, a hydrophobic core close to the future position of Mn1, involving Val173, Phe176 and Ile169 on helix B, Phe258, Ile262 on helix E and Leu236 and Leu240 on helix D is disrupted as helix D moves to a new position between helices E and G, where a number of new nonpolar and H-bonding interactions stabilize the new conformation. Finally, the C-terminal end of helix D (residues 239–245) unwinds from regular α-helix to 3_10_ helix and loop (Fig. S3).

The initial trigger for the conformational rearrangement is unclear, but it could be due to the binding of either one or both metal ions. Mn2 would force expulsion of the hydrophobic Val173 from the metal site, while Mn1 would stimulate the relatively large conformational changes seen in its ligand Glu170, the last residue before the refolding segment. However triggered, the conformational changes clearly open up a channel from the surface of the protein that leads to the Mn site, which has previously been proposed to be important for the diffusion of superoxide [10]. This channel is absent in the apo form of *L. blandensis* NrdB. The conformational changes are consistent with the observation that soaking crystals of the apo form with Mn^2+^ resulted in loss of diffraction, presumably due to changes in crystal packing. In seeming contradiction, Rose et al. have reported that the metal-loaded form of *F. johnsoniae* NrdB was generated by soaking apo crystals with Mn^2+^-containing solutions [10]. Thus, the crystal packing of *F. johnsoniae* NrdB could be such that the conformational changes can be accommodated. However, no apo structure of *F. johnsoniae* NrdB has been presented, so this remains speculative at present.

### Isolated *F. ignava* NrdB as well as holoenzyme contains an Mn_2_(IV,III) radical cofactor

**Fig. 5:**
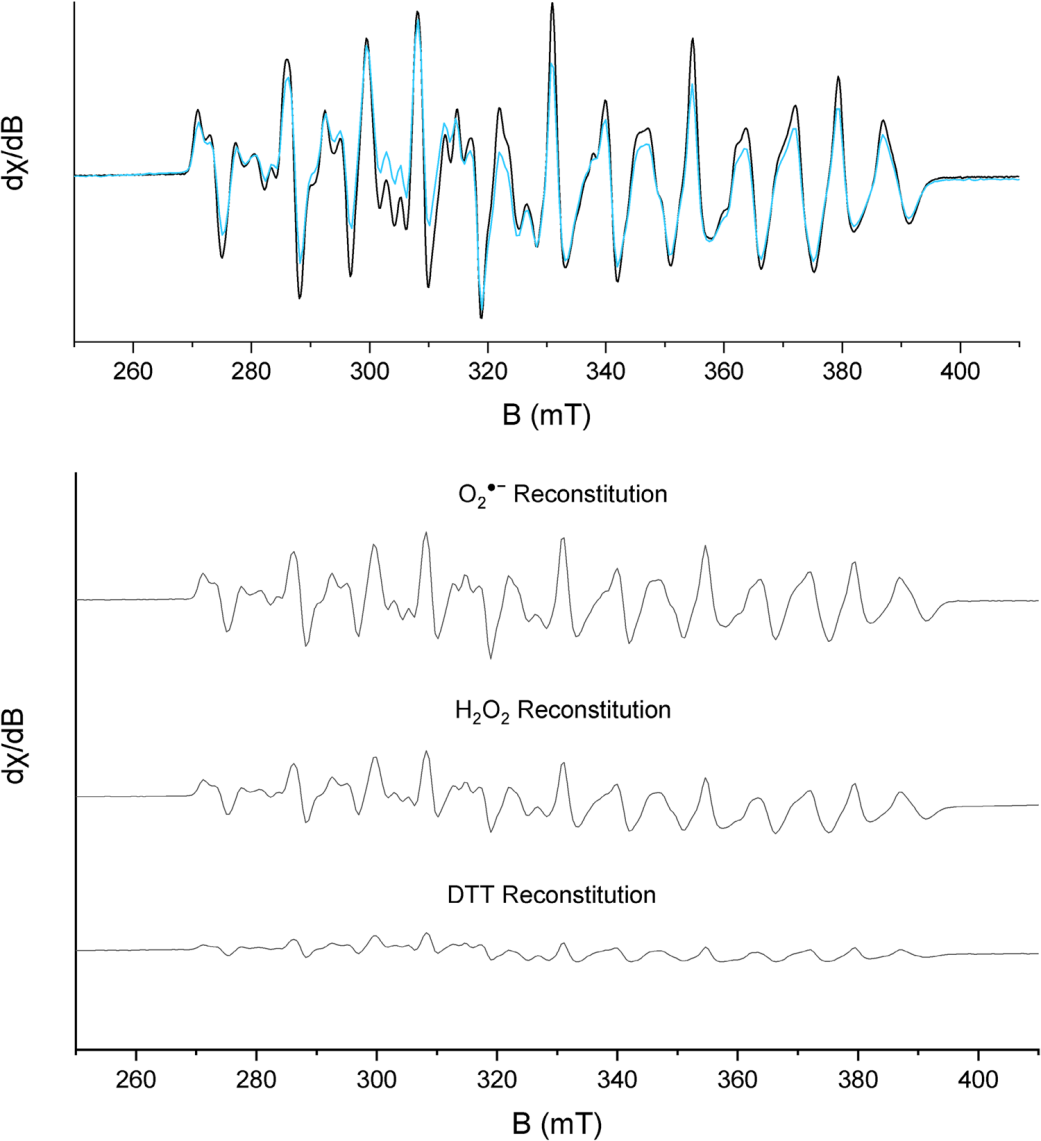
X-band EPR spectra of the metal cofactor in *F. ignava* NrdB in isolation and in the catalytic complex with NrdA, recorded on as-purified and in vitro reconstituted samples. Top: comparison between the isolated NrdB subunit (β_2_) and the active enzyme complex (α_2_β_2_). *Black spectra:* NrdB∆169^Mn^ (27 µM). *Blue spectrum*: NrdB∆Grx^Mn^ in complex with NrdA (NrdB∆Grx^Mn^ 27 µM, NrdA 100 µM, Mg(CH_3_CO_2_)_2_ 20 mM, ATP 2 mM). Bottom: various modes of in vitro reconstitution of the Mn_2_(IV,III) cofactor in NrdB. *O*_*2*_^·−^*reconstitution*: NrdB∆169^apo^ (100 µM), Mn^2+^ (200 µM), hydroquinone (200 µM). *H*_*2*_*O*_*2*_*reconstitution:* NrdB∆169^apo^ (100 µM), Mn^2+^ (200 µM) H_2_O_2_ (20 mM). *DTT reconstitution:* NrdB∆169^apo^ (25 µM), NrdA (100 µM), Mn^2+^ (50 µM), Mg^2+^ (40 mM), KCl (10 mM), ATP (3 mM), DTT (10 mM). EPR spectra collected at microwave frequency: 9.28 GHz; modulation amplitude: 10 G; microwave power: 1 mW; *T* = 10 K

The nature of the manganese cofactor in *F. ignava* NrdB was explored using X-band EPR spectroscopy. Low-temperature spectra revealed a complex multiline signal, which varied in intensity in a uniform fashion across the entire studied temperature (5–30 K, Figs. 5, S4) and microwave power range (1 µW–20 mW, Fig. S5) both for NrdB∆169^Mn^ as well as in vitro reconstituted samples (for details see below). At a microwave power of 1 mW, the highest signal intensity was observed at 10 K, and at *T* ≥ 30 K, no signal was observable. Importantly, no other signals were observed, suggesting a high degree of sample homogeneity. The shape, linewidth (125 mT) and peak split of the signal are in good agreement with an anti-ferromagnetically coupled Mn_2_(IV,III) cofactor, where the complex line shape is a result of a *S* = ½ system where the unpaired electron is interacting with two *I* = 5⁄2 Mn centres [25]. No tyrosyl radical signal could be seen in the collected EPR spectra, indicating that the tyrosyl radical equivalent electron hole is stored on the metal ions themselves.

The electronic structure of the manganese cofactor was also studied in *F. ignava* NrdB∆Grx in the presence of NrdA, ATP and Mg^2+^. As the NrdB∆Grx protein is more stable than the wild-type protein, it could be obtained in higher concentrations, suitable for EPR analysis, while still retaining the ATP-cone. Similar to the wild-type protein, this mutant, grown in LB medium supplemented with manganese, contained 1.76 mol Mn per NrdB monomer and its measured specific activity was 2226 nmol dCDP formed/mg min. Importantly, neither the presence of the ATP-cone nor the interaction with NrdA had any discernible effect on the shape of the multiline signal (Fig. 5 top panel, *blue spectrum*). As in the case of the isolated NrdB protein, the signal changed in a uniform fashion as a function of microwave power, but the power saturation curves displayed a noticeable shift towards higher *P*_1/2_ values (Fig. S6). In combination, this further supports the relevance of the Mn_2_(IV,III) cofactor as it is retained in the active enzyme complex, and suggests a stronger magnetic interaction between the NrdB metal cofactors in the active RNR complex (α_2_β_2_) as compared to the isolated NrdB dimer (β_2_).

### The Mn_2_(IV,III) cofactor is formed under aerobic conditions in the presence of a reductant

The in situ generation of active *F. ignava* RNR upon the addition of Mn^2+^ to NrdB∆169^apo^ in enzyme assay mixtures shows that the cofactor is spontaneously generated during these conditions, i.e. in the presence of air, ATP, Mg^2+^ and DTT. Consequently, a series of experiments was performed to elucidate the nature of the activating species. More specifically, samples of NrdB∆169^apo^ were prepared under conditions analogous to those used in the enzymatic assays, albeit at higher enzyme and metal concentrations, and the influence of oxygen as well as the chemical reductant DTT on the formation of the oxidized Mn cofactor was monitored by EPR spectroscopy.

The iron-dependent metal centres found in Fe_2_-containing class Ia as well as FeMn-containing Ic are oxidized by O_2_ to spontaneously form the active high-valent cofactor [13, 26]. However, the corresponding reactivity was not observed in *F. ignava* NrdB$\Delta$169. The addition of Mn^2+^ to NrdB did not result in significant assembly of the Mn_2_(IV,III) cofactor, and only a weak 6-line spectrum, attributable to mononuclear divalent Mn species, was observed following incubation on a timescale of several hours under aerobic as well as anaerobic conditions (Fig. S4). Likewise, the addition of Fe^2+^ ions to NrdBΔ169^apo^ either in isolation or in combination with Mn^2+^ did not result in any new EPR signal in the g $\approx$ 2 region (data not shown). Thus, assuming that the Fe-inhibited form incorporates Fe ions in the metal site, this species appears to be a diamagnetic, EPR silent, complex (e.g. Fe_2_(III,III)). Conversely, when DTT was added to the incubation mixture in combination with Mn^2+^ the formation of the Mn_2_(IV,III) cofactor was clearly visible from the appearance of the multiline EPR signature after aerobic incubation (Fig. 5 bottom panel, spectrum DTT Reconstitution). Spin quantification shows that this generates a signal with an intensity ≤ 40% of the NrdB$\Delta$169^Mn^ reference samples. It is noteworthy that formation of the Mn_2_(IV,III) species was observed also with trace amounts of oxygen in the solution. Consequently, overnight incubation of the protein samples in an anoxic atmosphere was required to completely avoid formation of the oxidized cofactor upon the addition of DTT. In combination, these experiments reveal that the high-valent cofactor is not spontaneously generated from oxygen. Rather, the mechanism is dependent on the combination of oxygen and reductant, possibly via in situ DTT-induced formation of reactive oxygen species (Eq. 1, note that the formation of water is not a strict requirement [27], the O_2_-derived O^2−^ ions could potentially be retained on the Mn cofactor) [28, 29]:

1 $$2 {\text{ Mn}}\left( {\text{II}} \right) \, + {\text{ O}}_{ 2} + {\text{ e}}^{ - } + {\text{ 4 H}}^{ + } \to {\text{ Mn}}_{ 2} \left( {{\text{IV}},{\text{III}}} \right) \, + {\text{ 2 H}}_{ 2} {\text{O}} .$$

The reactivity of the Mn(II)-loaded form of NrdB∆169 (Mn_2_(II,II)–NrdB∆169) towards reduced O_2_ derivatives was further studied via either in situ generation of O_2_^·−^ from hydroquinone (HQ) as previously described [10], or addition of aqueous H_2_O_2_. Treating Mn_2_(II,II)–NrdB∆169 with stoichiometric or slight excess of H_2_O_2_ had negligible effect on the metal centre, as determined from the very limited changes observed by both UV/Vis and EPR spectroscopy (data not shown). However, upon addition of 20 mM H_2_O_2_ to a 100 µM solution of Mn_2_(II,II)–NrdB∆169, the solution became light brown indicating the formation of a high-valent manganese species, with concomitant gas evolution. In parallel experiments, performed under similar conditions, the observed gas evolution was verified as O_2_ formation using a standard Clark electrode (data not shown). This indicates an H_2_O_2_ disproportionation activity of the NrdB subunit similar to that of manganese catalase, which cycles between Mn_2_(II,II) and Mn_2_(III,III) states during catalysis [30]. Still, EPR samples collected after 2-h incubation of Mn_2_(II,II)–NrdB∆169 in the presence of large excess H_2_O_2_ revealed the formation of a significant fraction of Mn_2_(IV,III)–NrdB∆169 (Fig. 5 bottom panel). The observed multiline signal was practically indistinguishable from the signal observed for NrdB∆169^Mn^ (Fig. 5 top panel), albeit the intensity was consistently lower (47 ± 10% at identical protein concentrations).

Similarly, treating solutions of *F. ignava* Mn_2_(II,II)-NrdB∆169 with 1 equivalent of HQ resulted in the formation of a light brown solution, and EPR spectra recorded on samples flash frozen 30 min after addition of HQ displayed a multiline signal identical to that observed for NrdB∆169^Mn^ and H_2_O_2_-treated Mn_2_(II,II)–NrdB∆169 (Fig. 5 bottom panel). However, it is noteworthy that O_2_^·−^ appeared significantly more efficient than H_2_O_2_ for the formation of the multiline signal. Spin quantification showed that the HQ-treated protein had a slightly higher EPR signal intensity than samples reconstituted with H_2_O_2_ (55 ± 5% relative to the NrdB∆169^Mn^ reference samples at identical protein concentrations) despite the fact that only equimolar amounts of HQ was used relative to Mn_2_(II,II)-NrdB, and no O_2_ formation was observed.

### NrdB activity correlates with Mn_2_(IV,III) cofactor content

**Fig. 6:**
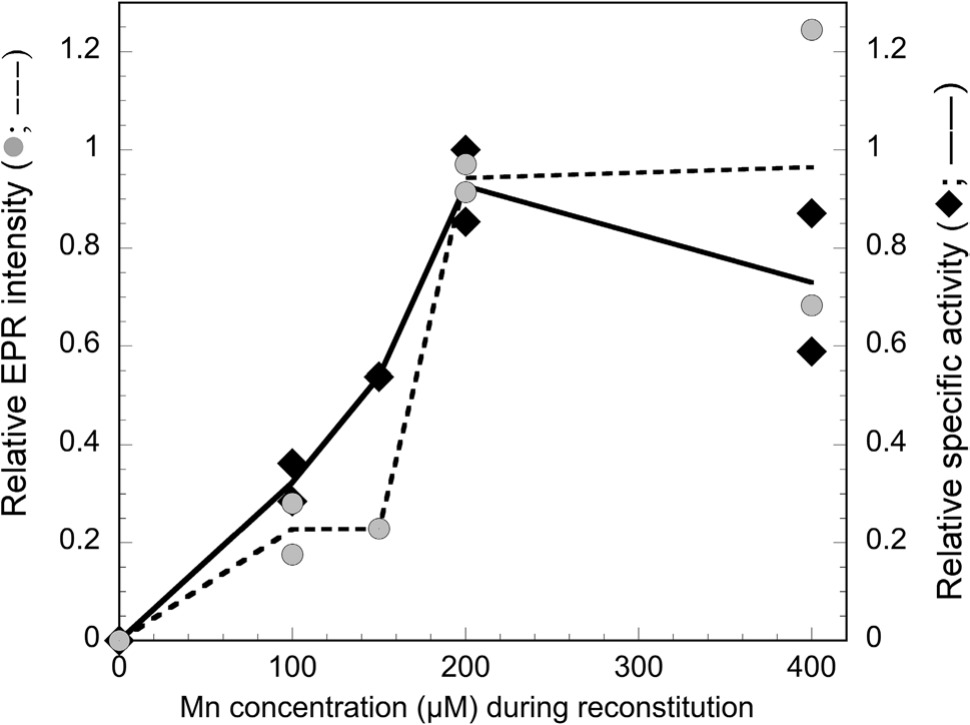
Correlation between intensity of Mn_2_(IV,III) multiline signal in NrdB∆169 and obtained catalytic activity. Two different sets of NrdB∆169^apo^ (200 µM monomer) samples were reconstituted aerobically with HQ and increasing Mn concentrations (0, 100, 200 and 400 µM in set 1, and 0, 100, 150, 200 and 400 µM in set 2), i.e. 2 mol Mn^2+^ per mol NrdB monomer at the highest Mn concentration. Each sample was desalted prior to EPR analysis and transferred to an anaerobic glove box for enzyme activity assays during anaerobiosis in an excess of Mn^2+^. The Mn concentration on the *X*-axis is the concentration of manganese added during reconstitution. Left axis, relative intensity of the multiline EPR signal of the Mn_2_(IV/III) metal cofactor (data points, grey circles; mean, dotted line); right axis, relative specific activity of EPR samples (maximal specific activity was 6476 nmol/min mg NrdB∆169^apo^; data points, black diamonds; mean, black line)

The elucidation of the conditions required to form the Mn_2_(IV,III) cofactor allowed us to specifically probe its relevance for the enzyme activity of NrdB, as compared to low-valent manganese ions. NrdB∆169^apo^ was reconstituted in vitro with increasing amounts of Mn^2+^ in the presence of HQ to generate samples with increasing amounts of the Mn_2_(IV,III) cofactor, followed by purification to remove any residual HQ and loosely bound low-valent metal ions. The relative amount of Mn_2_(IV,III) cofactor was determined in the different preparations by double integration of their respective multiline EPR signal. In parallel, aliquots from the desalted preparations were used in enzyme assays that were performed in the presence of excess Mn^2+^, but under strictly anaerobic conditions to prevent additional in situ formation of any high-valent manganese species. This ensured that the assay solutions contained practically identical concentration of Mn ions per NrdB protein, but varying amounts of the Mn_2_(IV,III) cofactor. No enzyme activity was observed for NrdB∆169 reconstituted in the absence of Mn^2+^ despite the presence of an excess of Mn^2+^ in the anaerobic assay mixture (Fig. 6). Conversely, the enzyme activity showed a strong correlation with the amount of Mn_2_(IV,III)–NrdB. The highest observed activity in these experiments corresponds to a *k*_cat_ of 8.8 s^−1^ for the enzymatically relevant dimeric form (β_2_) of NrdB∆169, and in agreement with the titration experiments shown in Fig. 1A. This peak activity was observed at an equimolar ratio of Mn^2+^ and NrdB monomer.

## Discussion

The data presented herein strongly support the dependence on a Mn_2_(IV,III) cofactor for efficient catalysis of *F. ignava* class I RNR. Its radical-generating subunit NrdB is clearly manganese dependent and high enzyme activity is obtained after expression in manganese-supplemented media, as well as in the metal titration assays. The crystal structure of the highly homologous NrdB protein from *L. blandensis* reveals a fully occupied dimeric manganese metal centre with Tyr207 (corresponding to *F. ignava* Tyr276 that aligns with the radical-containing tyrosine residue in RNRs from subclasses Ia and Ib) positioned with its −OH group at a distance of 7.6 Å from Mn1 in the metal site (Fig. 3), locked in that position by an H-bond to the conserved Thr294 (Thr363 in *F. ignava* NrdB). Albeit the crystal structure most likely reflects a low-valent form of the cofactor, the distance between the metals and Tyr207 appears to preclude the formation of a standard Mn_2_(III,III)-Y^·^ cofactor in class Id. Instead, enzymatically active NrdB reveals an intense multiline EPR signal attributable to a Mn_2_(IV,III) species, and there is no trace of any other EPR-active species, neither organic radical nor metal based (Fig. 5).

Similar Mn_2_(IV,III) species have been observed in earlier studies of class Id RNR, albeit always in combination with other EPR active species. Additionally, the in situ activation of the Id enzyme during standard assay conditions has prevented well-defined correlations between spectroscopically determined cofactor content and enzyme activity [10–12]. In this study, we have clearly shown that the enzyme activity correlates specifically with the Mn_2_(IV,III) cofactor content rather than total manganese content. Importantly, the spectrum observed for the catalytically competent NrdA:NrdB complex is practically indistinguishable from that of the isolated NrdB protein, arguing against the possibility of structural rearrangements resulting in the stabilization of a tyrosyl radical during holoenzyme formation.

In the context of metal usage, it is also noteworthy that the dimeric nature of the NrdB protein appears to have a significant influence, which is still not fully understood. *F. ignava* NrdB∆169 expressed in media supplemented with manganese co-purifies with 3.6 Mn per NrdB dimer, implying that the metal centres in both monomers are fully occupied, as is also evident from the crystal structure of the Mn-loaded form. Nevertheless, a saturation of enzyme activity occurs already when two Mn ions per dimer have been added to the apo-protein (i.e. one Mn^2+^/NrdB monomer), as evident from both the metal titration experiments (Fig. 1a) and reconstitution of the metal centre with Mn and superoxide (Fig. 6). Filling of the second metal centre with manganese does not appear to provide higher enzymatic activity. It is possible that two Mn_2_(IV,III) cofactors cannot be formed on one NrdB dimer. Similar observations have been made in earlier RNR studies, suggesting that only one tyrosyl radical forms per NrdB dimer [31–34]. Moreover, structures of Mn-dependent class Ib RNRs in *S. typhimurium* and *B. subtilis* reveal that the active RNR complex contains an asymmetric β_2_ dimer in solution, in which only one radical-generating subunit interacts with the α_2_ subunit and only one side of the tetramer is likely engaged in radical transfer [35, 36]. Likewise, in yeast RNR, NrdB is a heterodimer in which only one subunit possesses a metal centre and a radical [37]. The second NrdB subunit lacks metal-binding residues and is suggested to structurally stabilize the active enzyme, but not to be involved in radical formation. Similarly, it appears likely that only one Mn_2_(IV,III) cofactor per *F. ignava* NrdB dimer is required for catalysis and a fully active enzyme, while the other Mn-loaded NrdB monomer serves a structural role. More studies are needed to verify this.

**Scheme 1:**
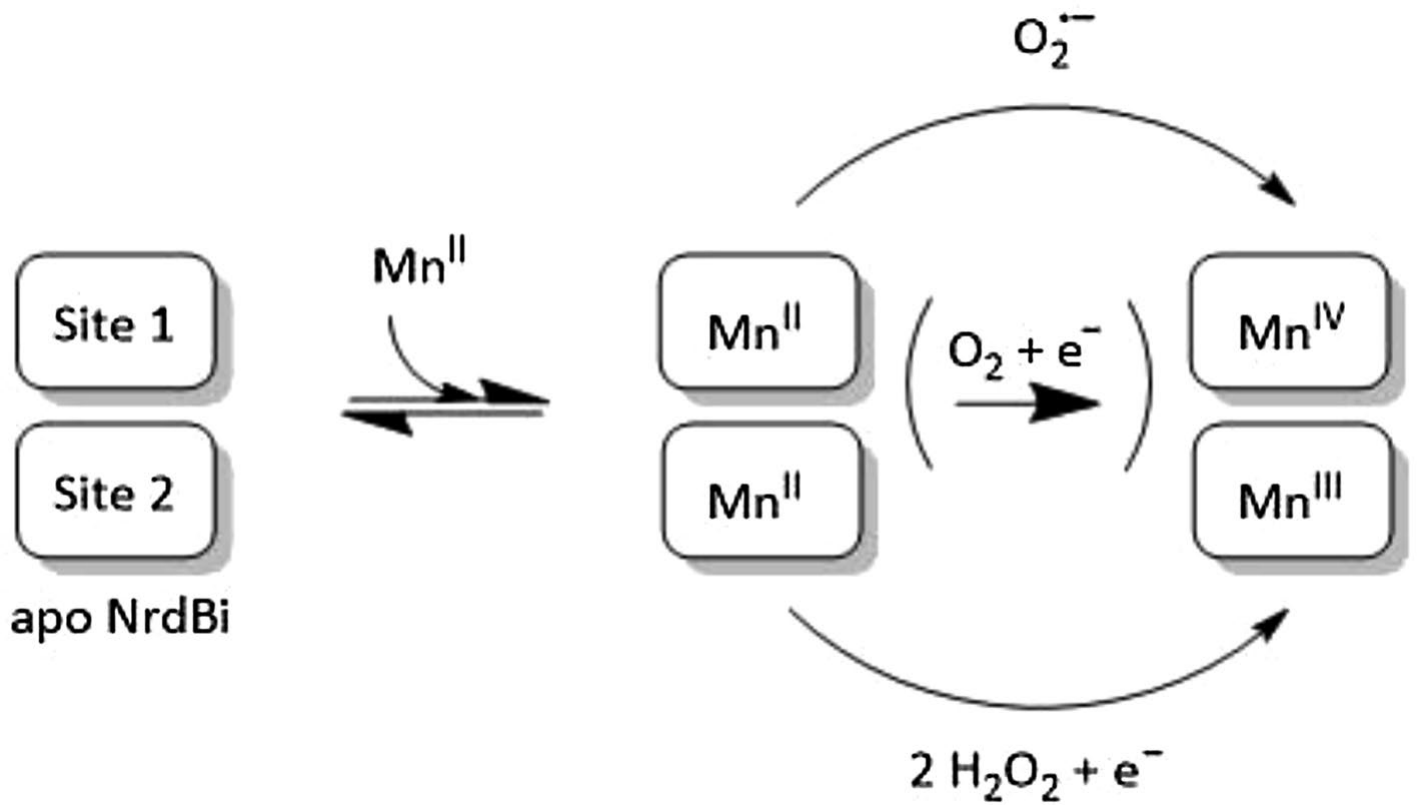
Schematic overview of the assembly pathways of the high-valent cofactor of class Id elucidated in this study. The assembly of the cofactor is initiated with the binding of two Mn(II) ions in the metal site of NrdB. The three-electron oxidation to form the active Mn_2_(IV,III) can then proceed through the reaction with (1) one equivalent of O_2_^·−^ or (2) two equivalents of H_2_O_2_ in combination with a one-electron reductant. A combination of these two pathways is likely to occur under aerobic conditions in the presence of a chemical reductant

In contrast to the Fe-containing cofactors found in class Ia and Ic RNRs, the high-valent manganese cofactor in class Id RNR does not form spontaneously under aerobic conditions, as expected from the intrinsic differences between Fe^2+^ and Mn^2+^ ions in their reactivity towards O_2_. Instead, the formation of the Mn_2_(IV,III) cofactor is dependent on the presence of reduced oxygen species, i.e. H_2_O_2_ or O_2_^·−^ (Scheme 1). Similar reactivity has been observed during the formation of the Mn_2_(III,III)-Y^·^ cofactor in class Ib, which proceeds via O_2_^·−^-induced oxidation of a Mn_2_(II,II) precursor [16, 38]. However, there are still a number of significant differences in reactivity between these two subclasses. In class Ib, the iron-loaded form (Fe_2_(III,III)-Y^·^) displays RNR activity, albeit considerably lower than the Mn_2_(III,III)-Y^·^ form [34, 39]. Conversely, iron does not appear capable of providing activity in subclass Id RNR, as no activity was observed when the enzyme was treated with Fe^2+^ ions in the absence of manganese, and neither our activity assays nor spectroscopy studies support the notion of a mixed metal (Fe,Mn) cofactor analogous to subclass Ic. Additionally, the activation of subclass Ib is strictly dependent on the flavodoxin-like protein NrdI, responsible for the formation and delivery of O_2_^·−^ through a well-defined channel in the quaternary complex between the NrdB subclass proteins denoted NrdF and NrdI [38, 40, 41]. No corresponding flavodoxin-like activase has been found for subclass Id. Instead extensive and unprecedented conformational rearrangements are observed when comparing the metal-free and metal-loaded forms of *Lb*NrdB, opening up a channel and exposing the metal site to the solvent.

Apo structures have been determined for RNR small subunits from *E. coli* (class Ia), *Corynebacterium ammoniagenes* and *Bacillus cereus* (class Ib), as well as *Chlamydia trachomatis* (class Ic) [20–23]. Class Ia and Ib proteins show rearrangement of the metal-binding ligands to occupy the space vacated by the metals, but there are no secondary structure changes. In *C. trachomatis* Ic, the situation is even more pronounced in that the metal ligands do not change conformation at all between apo and holo states. The larger structural changes seen in class Id *Lb*NrdB appear to be driven by metal loading, as binding of either Mn1 or Mn2 would be incompatible with the apo conformation and would encourage a change towards the Mn-containing form. The metal site is completely occluded in the apo state, thus metallation would require some proportion of the population to exist in a more open form that would allow Mn^2+^ to diffuse in and occupy one or more of the sites, driving the equilibrium towards the more open form. This highly solvent-accessible open form is then likely to facilitate the reactivity towards dissolved reactive oxygen species. Since all of the key residues are conserved within the subgroup of NrdBi that contains the equivalent of Lys174 in *Lb*NrdB, it seems safe to assume that similar differences would be observed in *F. ignava* NrdB and the whole subgroup. The NrdBi proteins that lack a conserved lysine at this position may adopt a different radical generation mechanism, but are still likely to carry a dimanganese cofactor as all metal ligating residues are conserved in the NrdBi subclass (Fig. 2). Potential differences in metal cofactor usage within NrdBi remains to be elucidated, as well as the in vivo assembly mechanism. Still, the data presented herein clearly show that class Id RNR utilizes a biochemically unique, stable Mn_2_(IV,III) cofactor to initiate catalysis. Moreover, based on the observed differences in efficiency between H_2_O_2_ and O_2_^·−^ in forming the high-valent cofactor, superoxide is arguably the physiologically relevant activating agent.

## Materials and methods

### General

All chemicals were purchased from Sigma-Aldrich or VWR and used as received unless otherwise stated. Cloning of the N-terminal hexa-histidine tag containing pET-nrdA, pET-nrdB, pET-nrdB∆169 and pET-NrdB∆Grx coding for *F. ignava* NrdA, NrdB, NrdB∆169 and NrdB∆Grx proteins was done as described in [12]. Cloning of pET-nrdB∆99 encoding *L. blandensis* NrdB∆99 (*Lb*NrdB∆99) was done as described in [11]. All anaerobic work was performed in an MBRAUN glovebox ([O_2_] < 10 ppm). Oxygen detection was done using a standard Clark electrode (Hansatech Instruments), separated from the sample solution by a Teflon membrane, and the signal was recorded using the CalMeter software package by Calmetric. Air-saturated water solutions ([O_2_]_25 °C_ = 253 µM) were used for the calibration of the electrode.

### Metal quantification by TXRF

The metal content of protein and buffer solutions was quantified using TXRF analysis on a Bruker PicoFox S2 instrument. A gallium internal standard at 2 mg l^−1^ was added to the samples (v/v 1:1) before the measurements. TXRF spectra were analysed using the software provided with the spectrometer. For each protein, measurements on three independently prepared samples were carried out. Average values and standard deviations are presented in Table 1.

### Protein expression

To overexpress the proteins, overnight cultures of *E. coli* BL21(DE3)/pET28a(+) bearing pET-nrdA, pET-nrdB, pET-nrdB∆Grx and pET-nrdB∆169 were diluted to an absorbance at 600 nm of 0.1 in LB (Luria–Bertani) liquid medium, containing kanamycin (50 µg/ml) and shaken vigorously at 37 °C. Isopropyl-β-d-thiogalactopyranoside (IPTG, Sigma) was added to a final concentration of 0.5 mM at an absorbance of *A*_600_ = 0.8. NrdB proteins were prepared from three different expression media: standard LB media (NrdB), standard LB media enriched with Mn^2+^ (NrdB^Mn^, NrdB∆169^Mn^), in which case standard LB media was supplemented with 0.5 mM Mn(CH_3_CO_2_)_2_ final concentration at the time of IPTG induction, and metal-depleted LB media (NrdB∆169^apo^) to which 0.5 mM EDTA was added before the induction. The cells were grown overnight at 30 °C and harvested by centrifugation. Overexpression of *L. blandensis* NrdB∆99 was performed in standard LB media (without the addition of metals) and was essentially as described above, except that the cells were grown overnight at 20 °C.

### Protein purification

Protein purification steps were done according to the protocols described in [11, 12]. The cell pellet was resuspended in lysis buffer: 50 mM Tris–HCl, pH 7.6, containing 300 mM NaCl, 10% glycerol, 10 mM imidazole, and 1 mM PMSF. For the purification of NrdB∆169^apo^, 0.5 mM EDTA was included in the lysis buffer. Cells were disrupted by high-pressure homogenization and the lysate was centrifuged at 18,000×*g* for 45 min at 4 °C. The recombinant His-tagged protein was first isolated by metal–chelate affinity chromatography using ÄKTA prime system (GE Healthcare): the supernatant was loaded on a HisTrap FF Ni Sepharose column (GE Healthcare) equilibrated with lysis buffer without phenylmethanesulfonyl fluoride (PMSF), washed thoroughly with buffer and eluted with buffer containing 500 mM imidazole. *F. ignava* NrdB∆Grx and NrdB∆169 were desalted on a Sephadex G-25 PD10 column (GE healthcare) equilibrated with buffer containing 50 mM Tris–HCl, pH 7.6, 300 mM NaCl, and 10% glycerol. *F. ignava* NrdA and NrdB and *L. blandensis* NrdB∆99 proteins were further applied to size-exclusion chromatography using a HiLoad 16/600 Superdex 200 pg column (GE Healthcare) using ÄKTA prime system, equilibrated with buffer containing 50 mM Tris–HCl, pH 7.6, 300 mM NaCl, and 10% glycerol. All buffers for the purification of *F. ignava* NrdA and full-length NrdB (but not the *F. ignava* NrdB∆Grx,NrdB∆169 and *L. blandensis* NrdB∆99) contained 2 mM DTT. *F. ignava* NrdA was further purified by hydrophobic interaction chromatography (HIC) as described in [12]. Protein concentrations were determined by measuring the UV absorbance at 280 nm based on protein theoretical extinction coefficients 99,700 M^−1^ cm^−1^ for *F. ignava* NrdA, 72770 M^−1^ cm^−1^ for *F. ignava* NrdB, 54320 M^−1^ cm^−1^ for *F. ignava* NrdB∆Grx, 51340 M^−1^ cm^−1^ for *F. ignava* NrdB∆169 and 39420 M^−1^ cm^−1^ for *L. blandensis* NrdB∆99. Protein purity was evaluated by SDS-PAGE (10–15%) stained with Coomassie Brilliant Blue. Proteins were concentrated using Amicon Ultra-15 centrifugal filter units (Millipore), frozen in liquid nitrogen and stored at − 80 °C until use.

### Metal site reconstitutions

All experiments were performed at room temperature and in 50 mM Tris–HCl, pH 7.6, 300 mM NaCl, 10% glycerol buffer. Unless otherwise stated, NrdB∆169^apo^ was used at a concentration of 100 µM in all experiments, and Mn(CH_3_CO_2_)_2_ concentrations ranged from 12.5 to 200 µM.

#### H_2_O_2_ assembly

NrdB∆169^apo^ and Mn(CH_3_CO_2_)_2_ were mixed and incubated for at least 30 min. Hydrogen peroxide was then added to a final concentration of 20 mM and incubated for at least 2 h.

#### O_2_^**·−**^ assembly

NrdB∆169^apo^ and Mn(CH_3_CO_2_)_2_ were mixed and incubated for at least 30 min. A stock solution of HQ (1 mM, solvated in aqueous buffer containing 0.1% ethanol) was then added to a final concentration of 1 mol HQ per mol monomeric NrdB and incubated for at least 30 min. For the metal cofactor concentration gradient, the designated substoichiometric amounts of Mn(CH_3_CO_2_)_2_ were added to NrdB∆169^apo^, and the assembly proceeded as described above.

### RNR activity measurements

#### Aerobic activity assay

RNR activity assays were performed as previously described [11]. Briefly, the reaction in a volume of 50 µl contained 50 mM Tris–HCl, pH 8, 10 mM DTT, 40 mM Mg(CH_3_CO_2_)_2_, 10 mM KCl, and 3 mM ATP. Mixtures of 0.25–1 µM NrdB or 0.25 µM NrdB∆169 and a tenfold excess of NrdA were used.

Prior to activity assays depicted in Fig. 1, indicated amounts of Mn(CH_3_CO_2_)_2_, or Mn(CH_3_CO_2_)_2_ in combination with Fe(SO_4_)·7H_2_O were mixed with 10 µM NrdB∆169^apo^ and incubated for 1 hour at room temperature on the bench without any addition of oxidant. The protein was then added into the reaction mixture to reach a final concentration of 0.25 µM. NrdA at a concentration of 2.5 µM was used.

Reactions were started by the addition of the CDP substrate to a final concentration of 0.8 mM. Enzyme reactions were incubated for 1–10 min at room temperature and then stopped by the addition of methanol. Substrate conversion was analysed by HPLC using a Waters Symmetry C18 column (150 × 4.6 mm, 3.5 µm pore size) equilibrated with buffer A (10% methanol in 50 mM potassium phosphate buffer, pH 7.0, supplemented with 10 mM tributylammonium hydroxide). Samples of 50–75 µl were injected and eluted at 0.4 ml/min at 10 °C with a linear gradient of 0–30% buffer B (30% methanol in 50 mM potassium phosphate buffer, pH 7.0, supplemented with 10 mM tributylammonium hydroxide) over 40 min for the separation of CDP and dCDP. Compound identification was achieved by comparison with injected standards. Relative quantification was obtained by peak height measurements in the chromatogram (UV absorbance at 271 nm) in relation to standards. Specific activities are given as nmol product formed per min and mg protein. From a direct plot of activity versus concentration of manganese, the apparent K_L_ values for binding of manganese to NrdB were calculated in SigmaPlot using the equation:

$$v\, = \,\left( {V_{ \hbox{max} } \cdot \left[ {\text{Mn}} \right]} \right)/\left( {K_{\text{L}} \, + \,\left[ {\text{Mn}} \right]} \right).$$

#### Anaerobic activity assay

The metal cofactor was assembled on NrdB∆169^apo^ with HQ as described above, and the excess superoxide, HQ and manganese were removed with a NAP-10 desalting column. The samples were then brought into an anaerobic glove box and equilibrated with the atmosphere overnight. The enzymatic assay mixture was prepared in PCR tubes as described above, with the addition of 1 mM Mn(CH_3_CO_2_)_2_. The NrdB∆169 concentration was 0.1 µM and NrdA 1 µM. The total concentration of NrdB∆169 was constant, but of that pool of protein only a certain fraction contained a Mn_2_(IV,III) cofactor, and the rest of the proteins contain no EPR-active manganese species. The CDP stock solution was prepared in a separate PCR tube. All PCR tubes were sealed in gas tight hypovials, and removed from the glove box. This way the assay could be performed in an anaerobic environment, on the bench. The enzymatic assay was started by the transfer of CDP from the CDP-vial to the assay mixture vials using a Hamilton syringe. The reactions were stopped by transfer of assay mixture into ice-cold 100% analytical grade methanol (on bench) using a Hamilton syringe. The product formation was measured as described in “Aerobic activity assay”.

### EPR spectroscopy

Measurements were performed on a Bruker ELEXYS E500 spectrometer using an ER049X SuperX microwave bridge in a Bruker SHQ0601 cavity equipped with an Oxford Instruments continuous flow cryostat and using an ITC 503 temperature controller (Oxford Instruments). Measurement temperatures ranged from 5 to 30 K, using liquid helium as coolant. The spectrometer was controlled by the Xepr software package (Bruker). EPR samples were frozen and stored in liquid nitrogen. The EPR spectra shown are representative signals from at least two individual experiments. Spin quantification was performed through double integration of the EPR spectra and calculated relative to NrdB∆169^Mn^. Unless otherwise stated, all spectra were recorded at 10 K, microwave power 1 mW, frequency 9.28 GHz, modulation amplitude 10 G and modulation frequency 100 kHz.

### Structure determination

#### Crystallization

The purified form of N-terminally truncated *L. blandensis* NrdB, lacking the ATP-cone domain, (*Lb*NrdB∆99) at 7.5 mg/ml in buffer containing 50 mM Tris–HCl, pH 7.8, 300 mM NaCl and 10% glycerol supplemented with 20 mM MgCl_2_ and 2 mM tris(2-carboxyethyl)phosphine (TCEP), was used for crystallization trials using commercial crystallization screens. An initial hit was obtained using the sitting drop vapour diffusion method with a protein to reservoir volume ratio of 200:200 nL equilibrated against 45 μl reservoir at 20 °C in a Triple Drop UV Polymer Plate (Molecular Dimensions, Newmarket, UK). A Mosquito nanolitre pipetting robot (TTP Labtech, UK) was used to set up the drops, which were imaged by the Minstrel HT UV imaging system (Rigaku Corporation, USA) at the Lund Protein Production Platform (LP3), Lund University. Crystals were obtained after 5-day incubation with a reservoir containing 2.4 M sodium malonate dibasic monohydrate, pH 7.0 (condition F9 of the JCSG Plus screen; Molecular Dimensions). Crystals were harvested directly from the drop, as the high concentration of sodium malonate present in the crystallization solution functioned as cryoprotectant. After failing to obtain data from apo crystals soaked with manganese, *Lb*NrdB∆99 protein was mixed with 10 mM MnCl_2_ and rescreened with the same commercial screens. Crystals of *Lb*NrdB∆99 with bound Mn^2+^ were obtained in 4 days with a reservoir containing 0.1 M Bis–Tris, pH 5.5, and 25% w/v polyethylene glycol 3350 (condition H10 of the JCSG Plus screen). Cryoprotectant consisting of reservoir solution with additional 20% glycerol was used.

#### Data collection and structure solution

The apo form of *Lb*NrdB∆99 crystallized in space group P2_1_2_1_2_1_ with unit cell dimensions 67.6 Å, *b* = 68.2 Å, *c* = 148.2 Å and one monomer in the asymmetric unit. Data were collected to 1.7 Å at EMBL Hamburg beamline P13 of the PETRA-III synchrotron using a Pilatus 6 M detector (DECTRIS). The diffraction images were integrated using XDS [42] and scaled using Aimless [43] from the CCP4 package [44]. The structure was solved by molecular replacement using Phaser [45] with a dimer of the NrdB protein from *Chlamydia trachomatis* [6] (PDB ID 1SYY, 23% sequence identity) as a search model. Non-conserved residues were pruned to the gamma carbon atom using CHAINSAW [46]. Phaser reported a solution with TFZ of 12.9 and LLG 171. Model extension was performed using Buccaneer [47, 48] and refinement was done with Refmac5 [49] (Table 1). This structure was rebuilt automatically in Buccaneer and manually in Coot [50], then refined to convergence using Buster (Global Phasing Ltd., Cambridge, UK).

The Mn-containing form of *Lb*NrdB∆99 crystallized in space group P2_1_2_1_2_1_ with unit cell dimensions *a* = 57.1, *b* = 80.6, and *c* = 158.2 Å and one dimer in the asymmetric unit. Data were collected to 1.84 Å at EMBL Hamburg beamline P14 of PETRA-III using an EIGER 16 M detector (DECTRIS, Baden-Daettwil, CH). Data were processed using XDS and scaled and merged using Aimless. The structure was refined using Buster. A further dataset was collected to 2.18 Å from the holo-form at a wavelength of 1.85 Å (high-energy side of the Mn^2+^ K edge at 1.8961 Å) to confirm the identity of the metal as Mn^2+^. Aimless estimated that there was a significant anomalous signal in this dataset to 2.83 Å. The holoenzyme structure was solved by molecular replacement with Phaser using the structure of one dimer of full-length *L. blandensis* NrdB (PDB 5OLK) with the ATP-cone removed. The coordinates were refined using Refmac [49] followed by Buster and manual rebuilding was carried out using Coot. For anomalous difference map calculation, the metal-loaded enzyme coordinates were refined using Buster against the dataset collected at 1.85 Å. Data processing and refinement statistics are presented in Table 2.

### Phylogeny

NCBI’s RefSeq database, downloaded in July 2018, was searched with subclass-specific HMMER [51] profiles for NrdBi and NrdBk plus an outgroup consisting of NrdBe and NrdBn used for RNRdb (http://rnrdb.pfitmap.org). The sequences were subsequently clustered at 70% identity with UCLUST [52] to reduce redundancy. After aligning the sequences with ProbCons [53] and selecting reliably aligned positions with BMGE [54] using the BLOSUM30 matrix, a matrix of 144 sequences and 158 aligned amino acids remained. This was used to estimate a maximum likelihood phylogeny with RAxML [55] using the PROTGAMMAAUTO model and rapid bootstopping. The alignment and phylogeny in nexml format were deposited at Figshare (10.17045/sthlmuni.8386652.v1). A phylogeny of the RNR class I NrdB proteins is shown in figure S1.

## Electronic supplementary material

Below is the link to the electronic supplementary material.

Supplementary material 1 (PDF 4547 kb)

**Supplemental Movie S1.** Animation illustrating the conformational changes between apo and metal-loaded states of *L. blandensis* NrdB∆99. A morph between the two states was generated using the rigimol option in MacPyMOL v.2.3.0 (Schrödinger LLC, NYC, USA). The intermediate states are only hypothetical and are not experimentally established. The movie has three phases: a) morph showing secondary structure elements only; b) morph showing all side chains as lines and the Mn ligands and other significant residues as sticks; c) morph with semi-transparent surface added to illustrate the opening of the channel to the metal site. (MP4 26869 kb)

## Abbreviations

RNR: Ribonucleotide reductase
WT-NrdB: Full-length *F. ignava* NrdB
NrdB∆Grx: *Facklamia ignava* NrdB protein lacking the 69-residue N-terminal Grx domain
NrdB∆169: *Facklamia ignava* NrdB protein lacking the N-terminal Grx and ATP-cone domains
*Lb*NrdB∆99: *Leeuwenhoekiella blandensis* NrdB protein lacking the N-terminal ATP-cone
Grx: Glutaredoxin
DOPA: 3,4-Dihydroxyphenylalanine
EPR: Electron paramagnetic resonance
DTT: Dithiothreitol
HQ: Hydroquinone
IPTG: Isopropyl β-d-1-thiogalactopyranoside
LB: Luria–Bertani broth
PMSF: Phenylmethylsulfonyl fluoride
TCEP: Tris(2-carboxyethyl)phosphine)
TXRF: Total reflection X-ray fluorescence
