# Supplementary material for: Class Id ribonucleotide reductase utilizes a Mn2(IV,III) cofactor and undergoes large conformational changes on metal loading
Source: J Biol Inorg Chem. 2019 Aug 14;24(6):863–77. doi: 10.1007/s00775-019-01697-8 (PMC6754362; doi:10.1007/s00775-019-01697-8)
Supplement: Supplementary file 1 — Supplementary material 1 (PDF 4547 kb) [file 775_2019_1697_MOESM1_ESM.pdf]

Class Id ribonucleotide reductase utilizes a Mn_2(IV,III) cofactor and undergoes large conformational changes on metal loading

Inna Rozman Grinberg[1], Sigrid Berglund[2], Mahmudul Hasan[3], Daniel Lundin[1], Felix M. Ho[2], Ann Magnuson[2], Derek T. Logan[3]*, Britt-Marie Sjöberg[1]*, Gustav Berggren[2]*

[1]Department of Biochemistry and Biophysics, Stockholm University, Sweden, [2]Department of Chemistry - Ångström Laboratory, Uppsala University, Uppsala, Sweden and [3]Department of Biochemistry and Structural Biology, Lund University, Lund, Sweden

Supplemental information

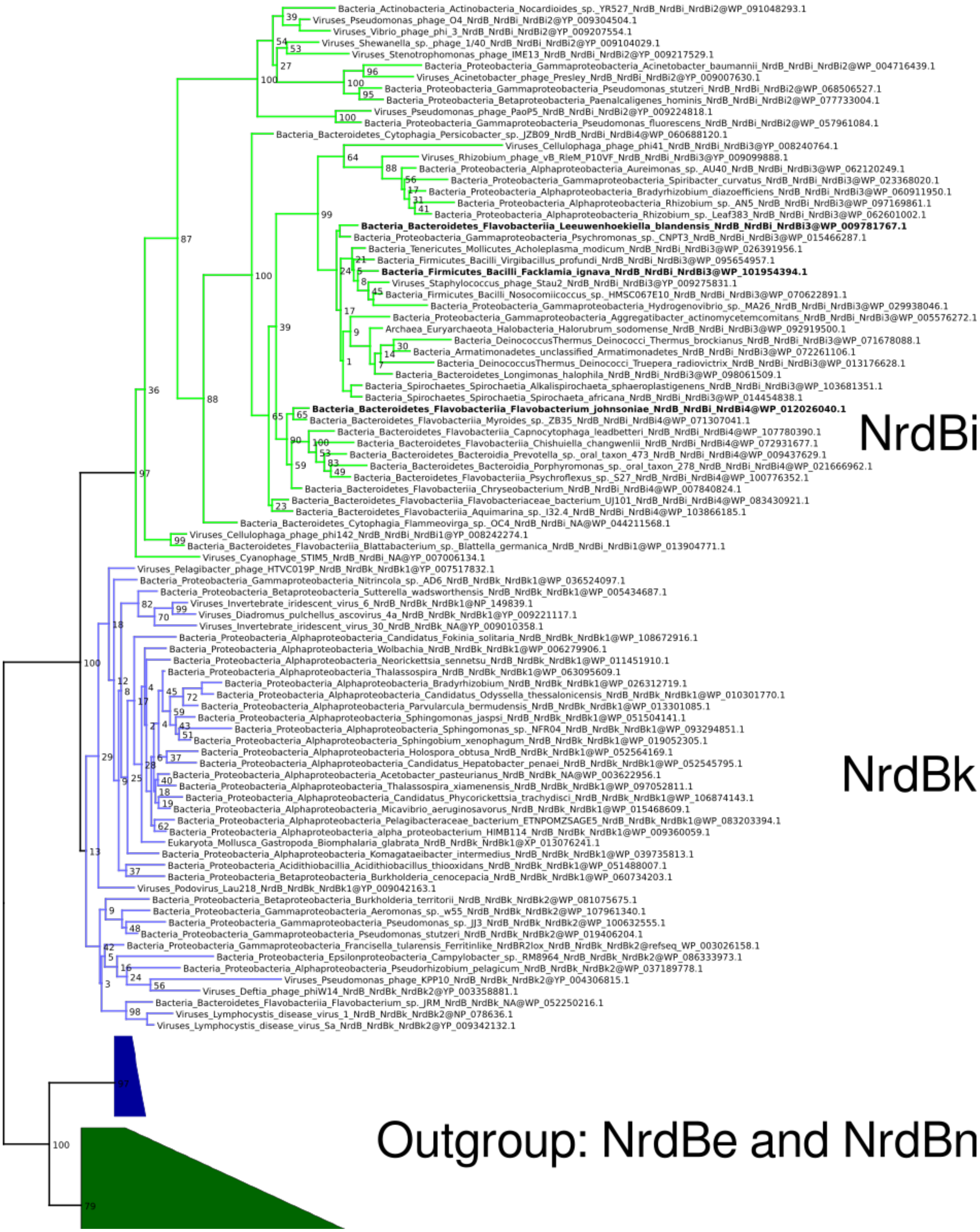

Supplemental Figure S1. Maximum likelihood phylogeny of NrdBi and NrdBk with sequences from NrdBe and NrdBn as outgroup. The outgroup was chosen based on relationships in the full NrdB phylogeny presented in [1]. For the full tree with outgroup in nexml format, see https://doi.org/10.17045/sthlmuni.8386652.v1.

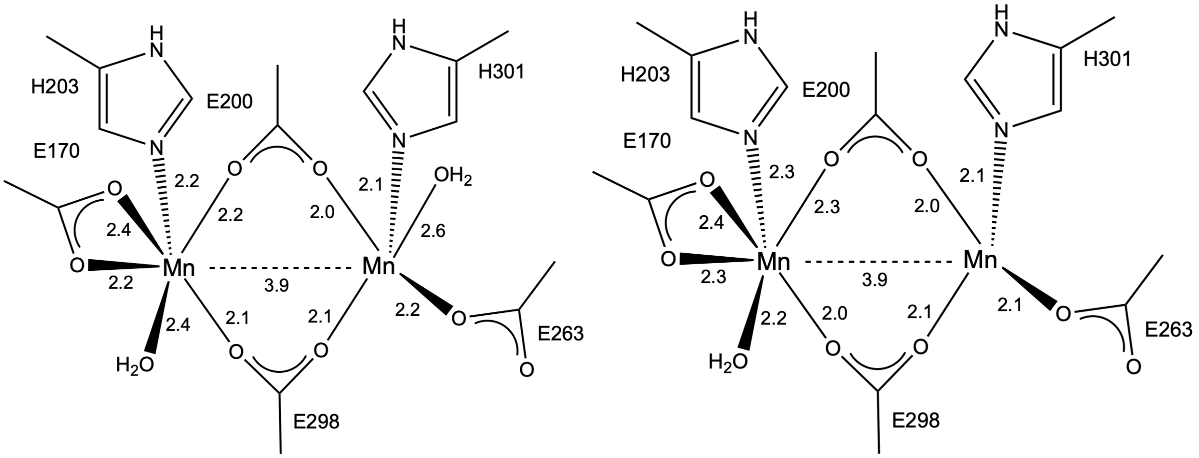

Supplemental Figure S2. Schematics of metal centers A and B in *L. blandensis* NrdBΔ99. Left: center A, Right: center B.

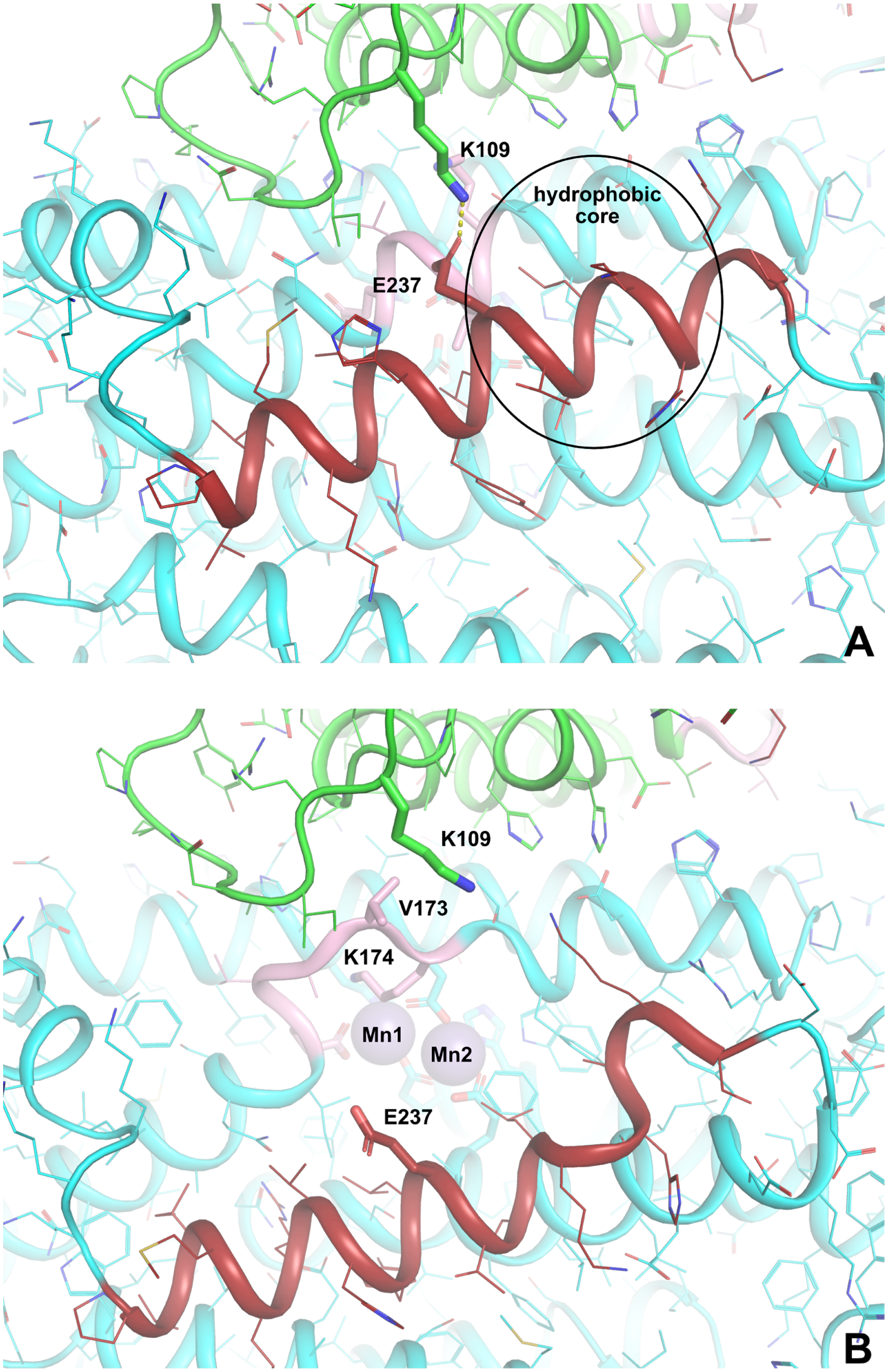


```
Helix B:
Lb: 153 evErsAikNtmLAIsQiEVaVKtFWgdvhhr 183
Fi: 222 phErtAirNamLAIsQvEVsVKtFWaklydr 252
Fj:  50 laEktAvkNslLAIaQiEVaVKsFWgniyeh  80
```

C

Supplemental Figure S3. Detailed picture of conformational changes between the apo and holo forms of *L. blandensis* NrdBΔ99. The proteins are drawn as cartoons and side chains are shown as lines, with significant amino acids highlighted as sticks. One monomer is colored light blue, with helix D highlighted in brick red and residues 170–174 in pink. The other monomer is colored green. Mn ions are drawn as spheres. A) Apo form; B) holo form. C) Sequence alignment of helix B in NrdB from *L. blandensis, F. ignava* and *F. johnsniae*. Mn ligating residue (*purple*), conserved residues specific to class Id NrdB (*red*).

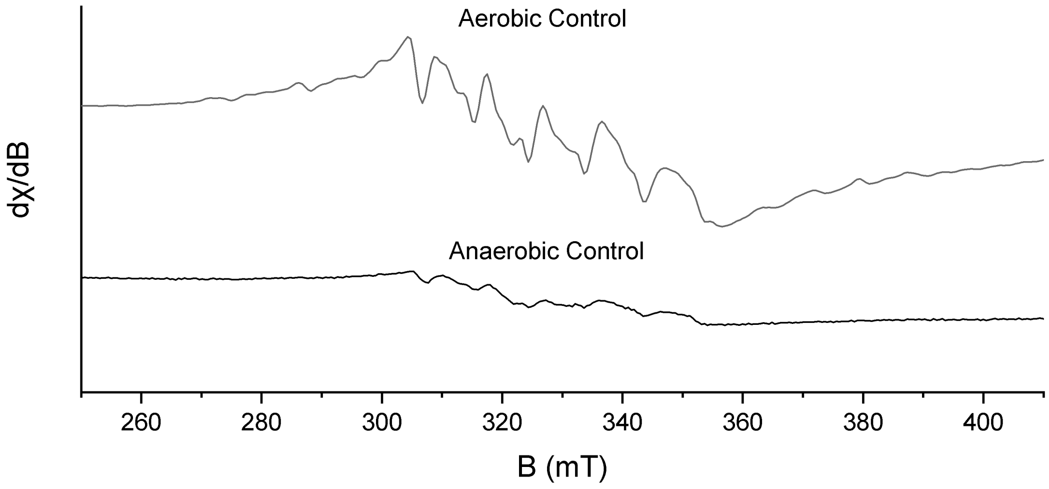

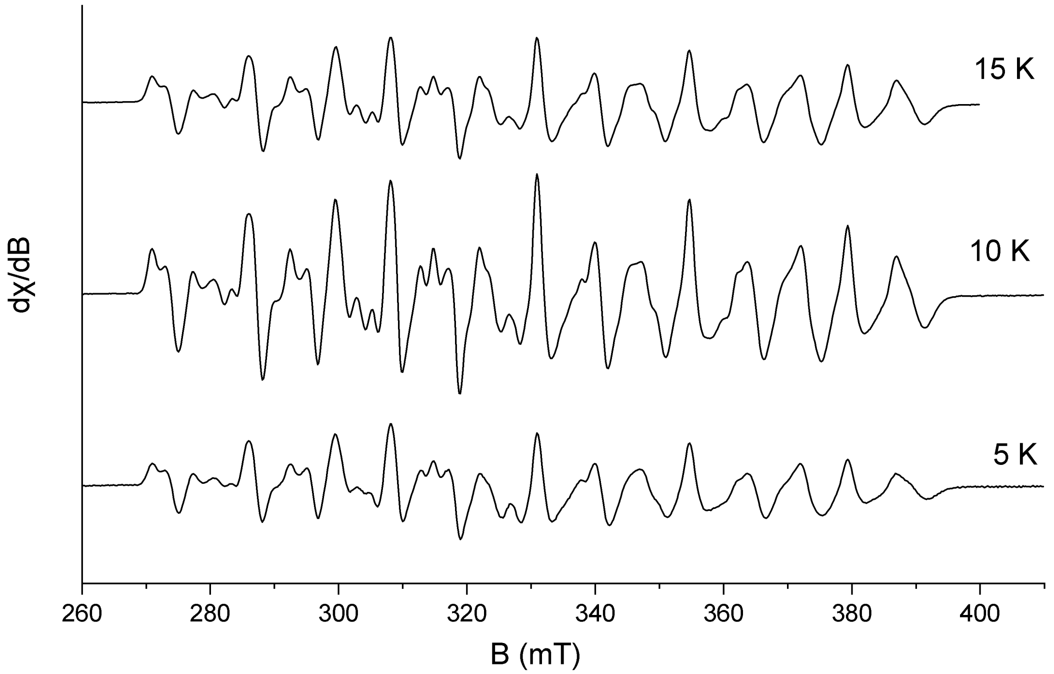

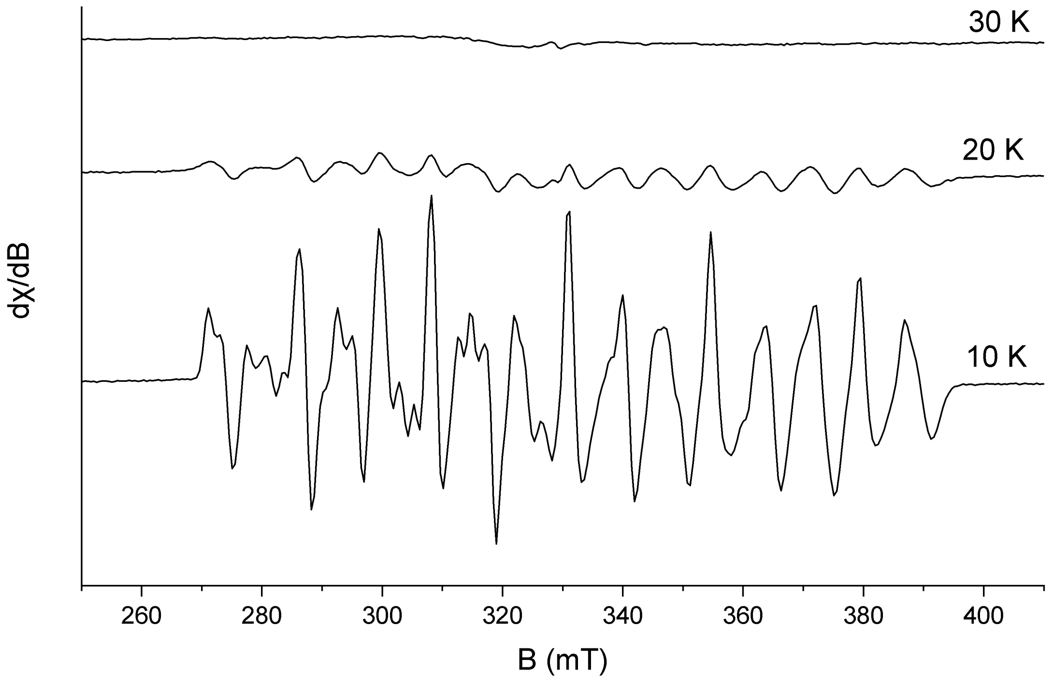

Supplemental Figure S4. **Top**: *Aerobic control:* NrdBΔ169^{apo} (100 μM), Mn^{2+} (200 μM). *Anaerobic control:* NrdBΔ169^{apo} (100 μM), Mn^{2+} (200 μM), under argon atmosphere. **Middle**: Temperature dependence of the EPR spectrum observed for as-purified NrdBΔ169^{Mn}. **Bottom**: Temperature dependence of the EPR spectrum observed for the as-purified and *in vitro* reconstituted samples *of* NrdBΔ169. EPR spectra collected at microwave frequency: 9.28 GHz; modulation amplitude: 10 G; microwave power: 1 mW; T = as indicated in the figure).

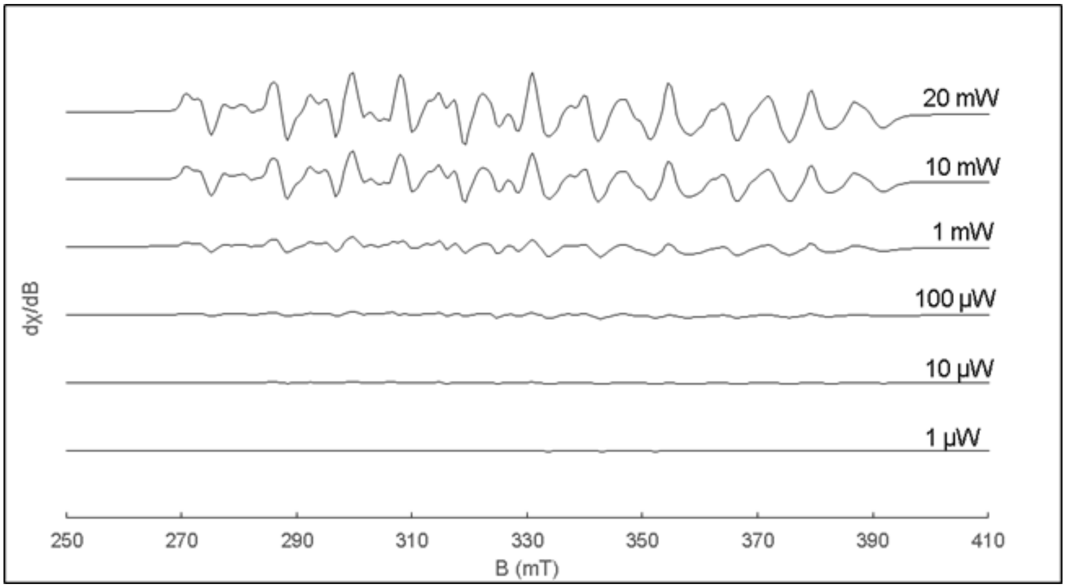
20 mW
10 mW
1 mW
100 μW
10 μW
1 μW
dχ/dB
250 270 290 310 330 350 370 390 410
B (mT)

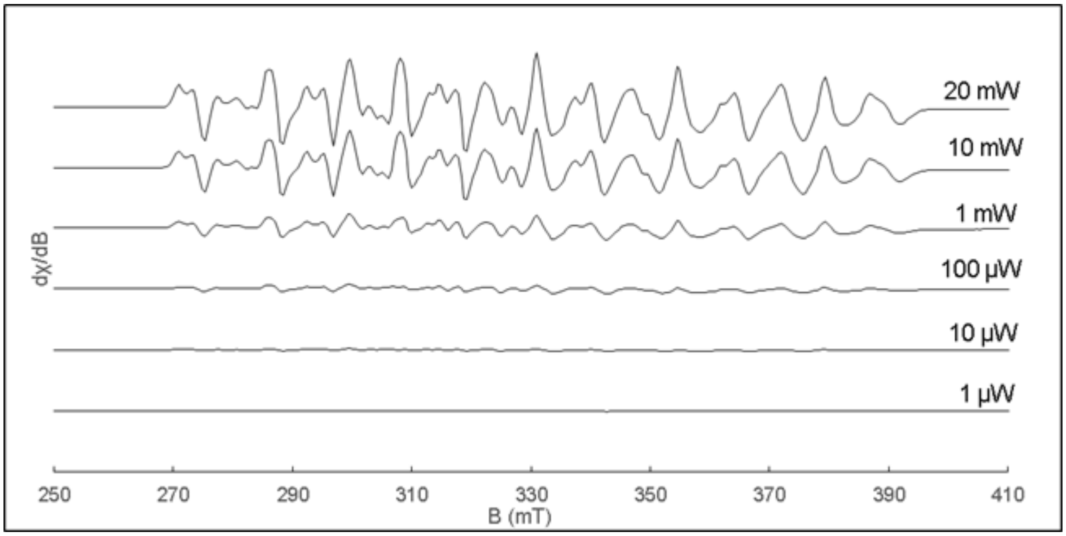
20 mW
10 mW
1 mW
100 μW
10 μW
1 μW
dχ/dB
250 270 290 310 330 350 370 390 410
B (mT)

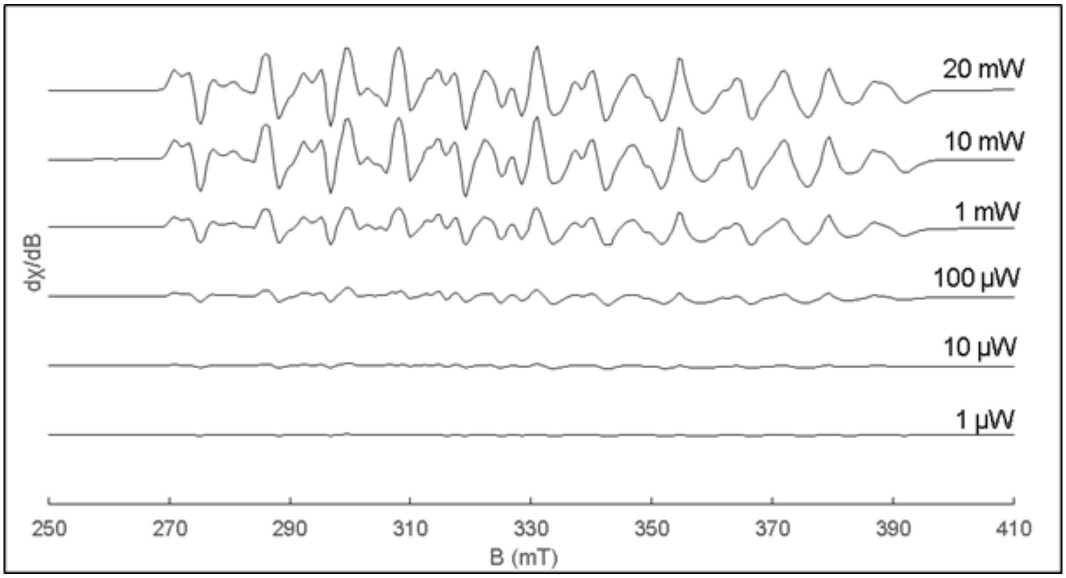
20 mW
10 mW
1 mW
100 μW
10 μW
1 μW
dχ/dB
250 270 290 310 330 350 370 390 410
B (mT)

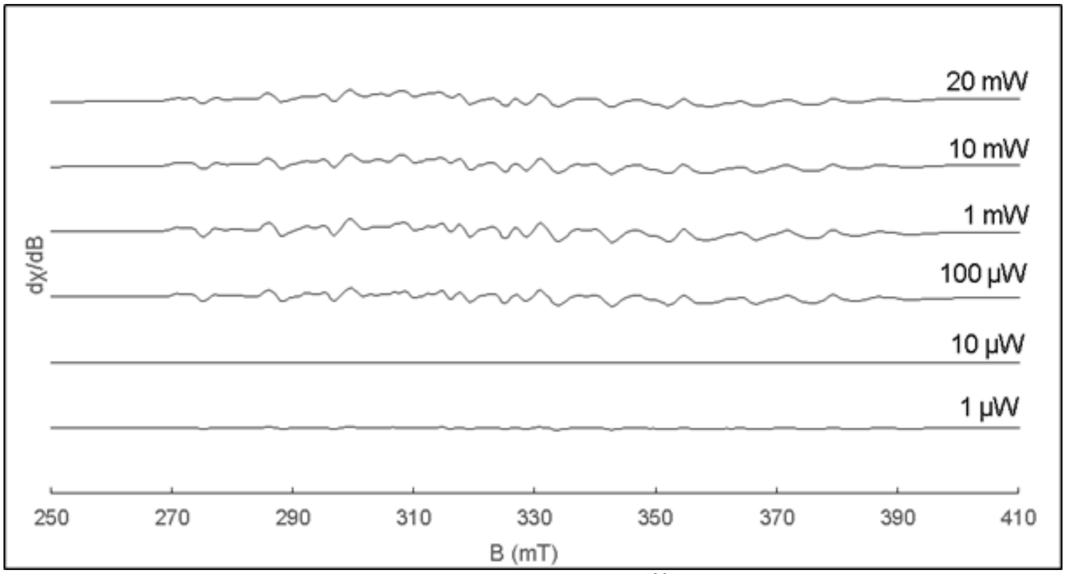

Supplemental Figure S5. EPR spectra of *F. ignava* NrdBΔ169^{Mn} over a range of microwave powers and temperatures, indicating the signal power dependence, and the spectral purity of the Mn_2(IV,III)-assigned 16-line signal. Microwave powers are indicated in each spectrum, spectra in each panel has been collected at one temperature. From top to bottom: 20 K, 15 K, 10 K, 5 K.

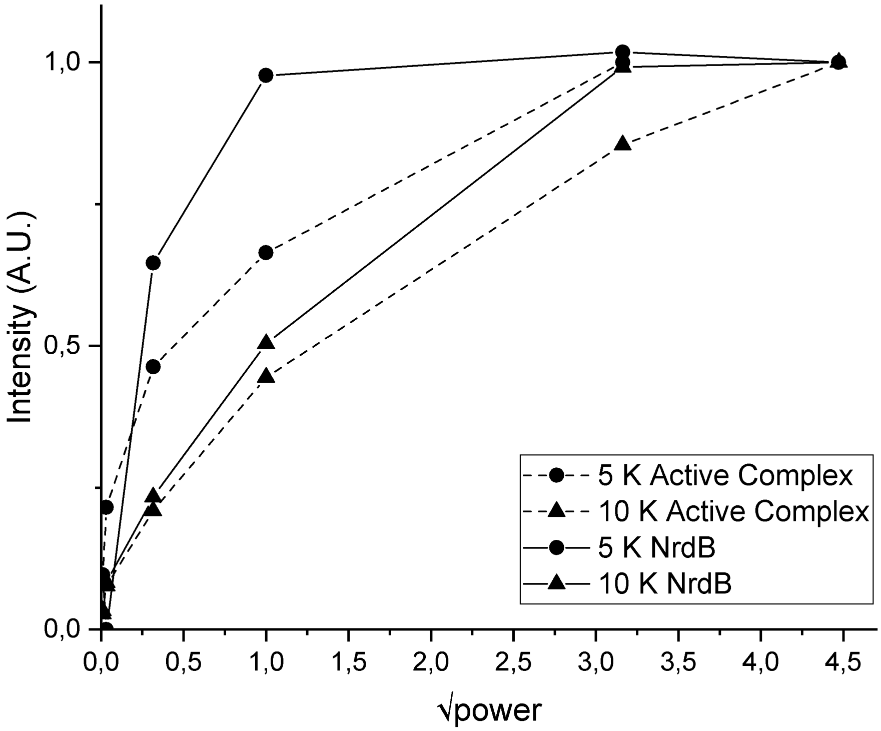

Supplemental Figure S6. EPR power saturation behaviour of the Mn_2(IV,III) cofactor in isolated *F. ignava* NrdBΔ169^{Mn} (β_2), solid lines and in the active RNR complex of NrdBΔGrx and NrdA ($\alpha_2\beta_2$), dashed lines. Individual P/T series normalized to highest intensity in each series for clarity. Power dependence of the proteins was studied at 5K (black circles) and 10K (black triangles), and estimated from the intensity

of the full spectra. Spectra collected at microwave frequency: 9.28 GHz; modulation amplitude: 10 G; A shift towards higher $P_{1/2}$ values is observed for the $\alpha_2\beta_2$ complex relative to β_2 at both temperatures.

References

1 I. Rozman Grinberg, D. Lundin, M. Hasan, M. Crona, V. R. Jonna, C. Loderer, M. Sahlin, N. Markova, I. Borovok, G. Berggren, A. Hofer, D. T. Logan and B. M. Sjöberg (2018) Elife 7:e31529
